# Comparative Efficacy and Acceptability of Endoscopic Methods for Rectal Neuroendocrine Neoplasms with Low Malignant Potential: A Network Meta-analysis

**DOI:** 10.5152/tjg.2024.23477

**Published:** 2024-06-01

**Authors:** Shun-Tao Zhang, Qi Chen, Yuan-Meng Zhang, Qiao-Yu Li, Yu-Chen Gao, Wen-Jun Meng, Lie-Wang Qiu, Bo Zeng

**Affiliations:** 1 Department of Gastroenterology, Yongchuan Hospital, Chongqing Medical University, Chongqing, China; 2 Department of Gastrointestinal Surgery, Yongchuan Hospital, Chongqing Medical University, Chongqing, China; 3 Department of Endocrinology, Zigong Fourth People’s Hospital, Zigong, China; 4 Department of Biotherapy, Cancer Center, West China Hospital, Sichuan University, Chengdu, China

**Keywords:** Rectal neuroendocrine neoplasms, colonoscopy, endoscopic mucosal resection, therapeutics, network meta-analysis, endoscopic submucosal dissection

## Abstract

**Background/Aims:**

Although endoscopic resection is an effective treatment of rectal neuroendocrine neoplasms (R-NENs) with low malignant potential, there is no consensus on the most recommended endoscopic method. This study aimed to assess the efficacy and acceptability of different endoscopic treatments for R-NENs with low malignant potential.

**Materials and Methods:**

We searched databases for studies on treatments of R-NENs using endoscopic resection. These studies comprised techniques such as endoscopic mucosal resection (EMR), endoscopic submucosal dissection (ESD), modified endoscopic mucosal resection (EMRM), modified endoscopic submucosal dissection (ESDM), and transanal endoscopic microsurgery (TEM). The primary outcomes assessed were histological complete resection (HCR).

**Results:**

Overall, 38 retrospective studies (3040 R-NENs) were identified. Endoscopic mucosal resection with a cap (EMRC), endoscopic mucosal resection with ligation (EMRL), ESD, ESDM, and TEM demonstrated higher resectability than did EMR in achieving HCR. Endoscopic mucosal resection, EMRC, EMRL, EMRP, EMRD, and EMRU required shorter operation times than did ESD. Endoscopic mucosal resection, EMRC, ESDM, and TEM incurred lower risks than did ESD.

**Conclusion:**

Regarding R-NENs ≤20 mm with low malignant potential, ESD could be used as the primary treatment. However, TEM may be more effective if supported by economic conditions and hospital facility. With respect to R-NENs ≤16 mm with low malignant potential, EMRL could be used as the primary treatment. In regard to R-NENs ≤10 mm with low malignant potential, EMRL, EMRC, and ESD could be used as the primary treatment. However, EMRL and EMRC might be better when operational difficulties and economic conditions were considered.

**Main Points**

- Regarding rectal neuroendocrine neoplasms ≤20 mm with low malignant potential, endoscopic submucosal dissection (ESD) outperformed endoscopic mucosal resection (EMR) in terms of resectability, whereas safety was a concern. Transanal endoscopic microsurgery (TEM) outperformed ESD in terms of resectability and safety, whereas surgery time and medical cost were concerns. Endoscopic submucosal dissection could be used as the primary treatment. However, TEM might be more effective if supported by economic conditions and hospital facility.
- In regard to rectal neuroendocrine neoplasms ≤16 mm with low malignant potential, endoscopic mucosal resection with ligation (EMRL) combined the resectability by ESD with the safety of EMR with shorter operative time and lower cost than ESD. Endoscopic mucosal resection with ligation (EMRL) could be used as the primary treatment.
- Considering rectal neuroendocrine neoplasms ≤10 mm with low malignant potential, EMRL, endoscopic mucosal resection with a cap (EMRC), and ESD showed better resectability and similar safety than did EMR, whereas EMRL and EMRC also demonstrated shorter time and lower cost than did ESD. EMRL, EMRC and ESD could be used as the primary treatment. However, EMRL and EMRC might be better when operational difficulties and economic conditions were taken into account.

## Introduction

Neuroendocrine neoplasms are a group of heterogeneous tumors that frequently occur in the gastrointestinal tract, particularly in the rectum. The incidence of rectal neuroendocrine neoplasms (R-NENs) accounts for approximately 20% of the total gastrointestinal neuroendocrine neoplasms.^1^ This incidence is constantly updated as preventive screening for colon cancer has gained increasing interest.^2^ Although early-stage R-NENs are less malignant and indicate good prognosis, the prognosis of progressive R-NENs was found to be similar to that of adenocarcinomas.^3^ Therefore, early diagnosis and treatment are very important.

The treatment approach for R-NENs depends on their malignant potential. According to the 2012 European Association of Neuroendocrine Neoplasms,^4^ endoscopic local excisional treatment is considered feasible for R-NENs ≤20 mm, well differentiated (G1-G2), and without lymphovascular involvement or invasion of the proper muscular layers. The pursuit of histologically effective resection has led to traditional polypectomy replaced with endoscopic mucosal resection (EMR) and endoscopic submucosal dissection (ESD). Modified EMR (EMRM) and modified ESD (ESDM) were developed to balance between the safety and resection capability of EMR and ESD. In addition, transanal endoscopic microsurgery (TEM) is increasingly used as the initial treatment of R-NEN, previously used as a salvage procedure for incomplete clearance of R-NENs.^5^ However, there is no consensus on the most appropriate type of endoscopic intervention. Previous meta-analyses have assessed the efficacy and acceptability of ESD versus EMRM and ESD versus EMR.^6,7^ However, these analyses did not provide an adequate reference due to the limited interventions included. Network meta-analysis (NMA) could provide the highest evidence for treatment guidelines,^8^ including a comparison of direct and indirect treatments, thereby providing more comprehensive recommendations for decision-making.

Particularly, exploring optimal endoscopic treatment modalities is important to increase the rate of early and effective treatments; improve patient survival; enhance the quality of patient care; and rationalize the use of healthcare resources. Therefore, this study compared the efficacy and acceptability of the existing endoscopic treatment modalities using an NMA to guide clinicians in developing optimal treatment strategies.

## Materials and Methods

This study adhered to the guidelines of the Preferred Reporting Items for Systematic Reviews and Meta-Analyses Extension Statement for Network Meta-Analysis (PRISMA-NMA; Supplementary Table 1).^9^ The study protocol was registered in the Prospective Register of Systematic Reviews (PROSPERO CRD42023417278).

### Search Strategy

Databases, including PubMed, Embase, Cochrane, CNKI, and Wanfang Data, were searched from January 2010 to March 2023 to retrieve relevant clinical studies. The following terms were used in combination (see Supplementary Table 2): “Rectal Neoplasms,” “Neuroendocrine Tumors,” “Carcinoid Tumor,” “Endoscopic Mucosal Resection,” and “Endoscopic Submucosal Dissection.” Additionally, we manually searched the reference lists for relevant publications. No language or geographic restrictions were imposed. The filtered results were then imported into the Endnote Library (version ×9.3) for management.

### Selection Criteria

To be eligible for this NMA, studies needed to meet the following criteria: First, adult patients underwent endoscopic therapies and were diagnosed with R-NENs after treatment. Second, endoscopic ultrasound or pathological examination suggested that R-NENs had low malignant potential (size ≤20 mm in diameter, well differentiation, no lymphovascular invasion, or invasion limited to mucosal or submucosal). Third, endoscopic techniques such as EMR, ESD, EMRM, ESDM, or TEM were included. Fourth, outcomes included histological complete resection (HCR). The main criteria for study exclusion were (i) duplicate publications, (ii) inaccessibility to original literature, (iii) non-clinical studies, and (iv) missing critical information to determine whether the inclusion criteria were met.

### Literature Selection and Quality Assessment

To ensure data extraction’s accuracy and research’s rigor, 2 researchers (S.Z. and Q.C.) independently extracted, integrated, and cross-checked the data, while assessing the methodological quality of each included original study. The Newcastle Ottawa Scale was used to determine whether they were high quality (score 8 or 9), medium quality (score 6 or 7), or low quality (score ≤5).^10^ Any disagreements regarding data extraction and quality assessment were resolved through discussion and judgment by a third investigator (B.Z.).

### Outcomes

The goal of treatment was to achieve complete histological resection. Therefore, the primary outcome events included HCR, which represented no residual tumor tissue confirmed by pathological examination after endoscopic resection. Additionally, surgery time and complications (including procedure-related bleeding and perforation) have also been a focus of attention.

### Statistical Analysis

Log odds ratios (OR) with a 95% confidence interval (CI) were used to compare binary outcomes. The mean difference (MD) and 95% CI were calculated for the continuous outcomes. The NMA was conducted using a random-effects Bayesian framework to predict the effects of all measures simply and straightforwardly.^11^ All direct and indirect evidence was combined to compare HCR, surgery time, and complication of various techniques for R-NENs. Subgroup analysis was performed, stratified by morphology (size ≤10 mm in diameter) and histology (low malignant potential confirmed by pathological examination). Meta-regression was performed to explore source of heterogeneity. The analysis was performed using the multinma package^12^ and getmc package^13^ in R (version 4.1.3). First, network diagrams were plotted to visualize the treatments compared directly or indirectly. Next, the log OR and MD of the pairwise comparisons were presented as league tables. The ranking probability of each measure was then calculated; a ranking curve was plotted. Heterogeneity among studies was assessed using the *I*^2^ statistic. Moreover, the prediction intervals were displayed in a forest plot. In addition, potential inconsistencies between direct and indirect evidence were assessed using the deviance information criterion and node-splitting method. A funnel plot was created to assess the potential bias due to the small sample size, using symmetry as an evaluation criterion.

### Assessment of Certainty of the Evidence

The final outcome reliability assessment of the NMA followed the guidelines of the Grading of Recommendations, Assessment, Development, and Evaluation (GRADE) Working Group.^14^ The GRADE approach classifies the quality of evidence into 4 levels: high, moderate, low, and very low. For retrospective studies with an initial quality of evidence rated as “‘low,” it was downgraded if issues of study bias (with high risk), reporting bias, indirectness, heterogeneity, or inconsistency were identified. Conversely, evidence was upgraded if there was a large magnitude effect in each pair comparison.

## Results

### Study Selection

**Figure 1.**
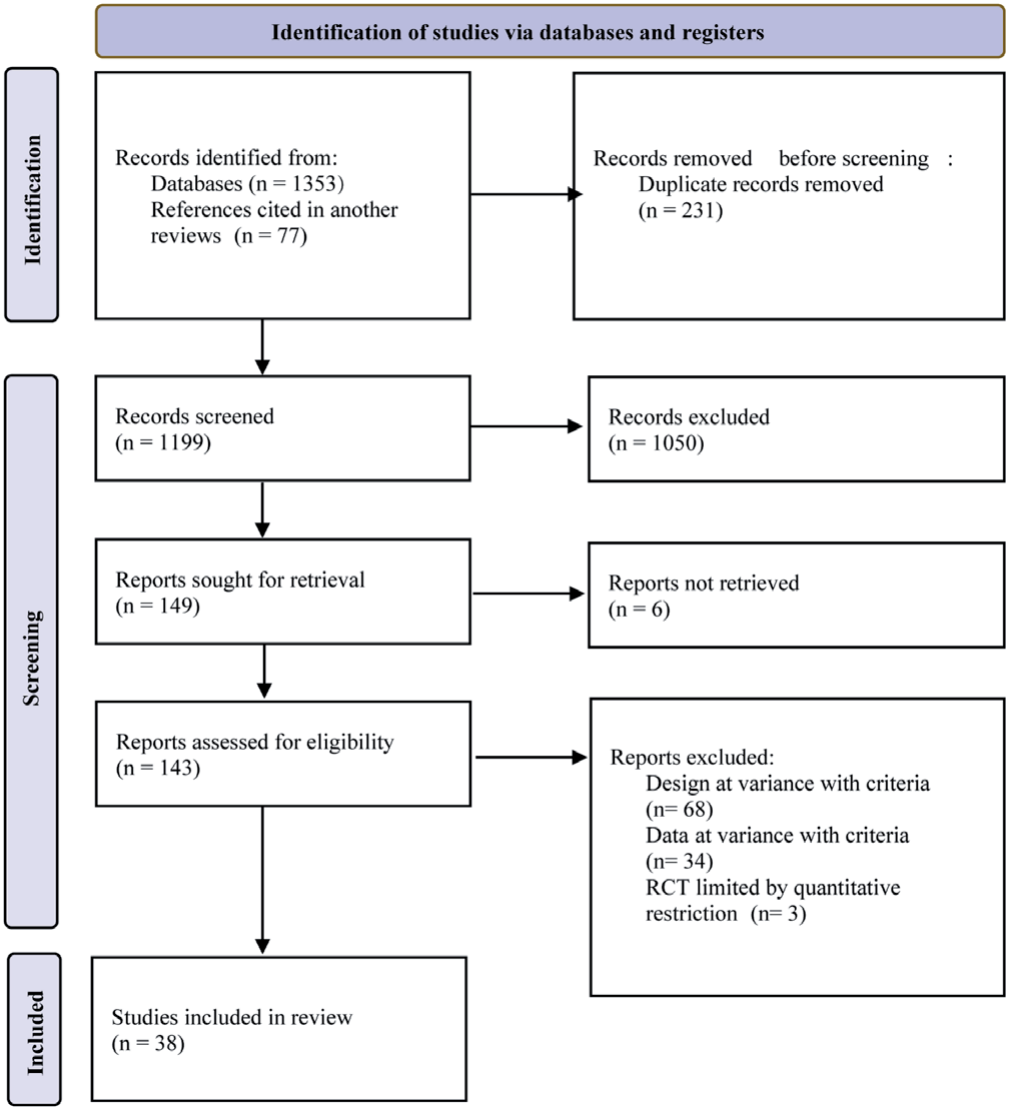
Research screening process.

**Supplementary Figure 1.**
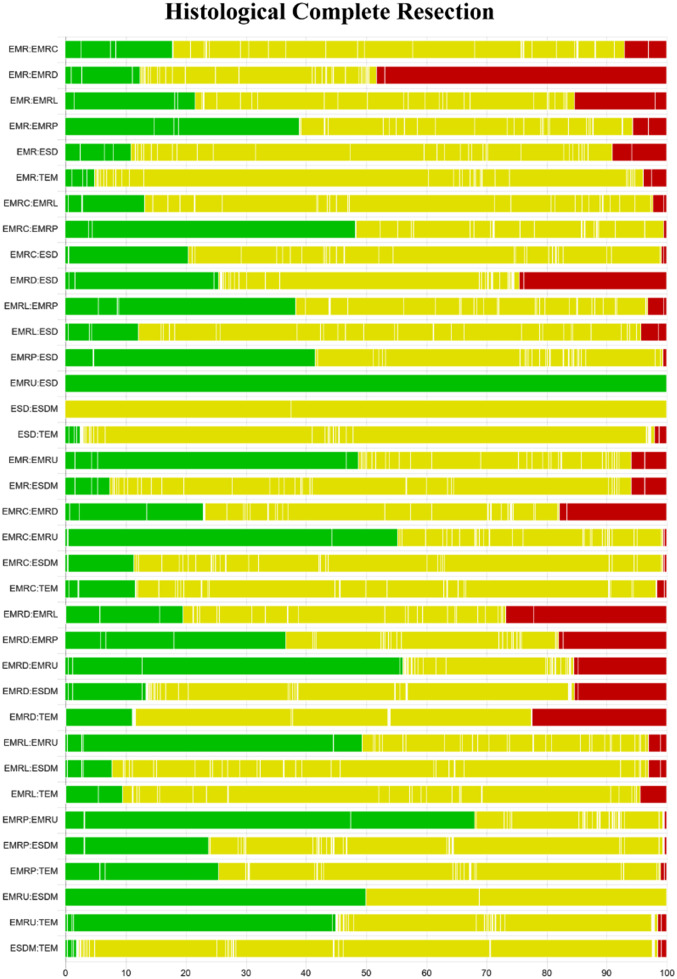
Quality assessment of pairwise comparisons for HCR. Comparisons on endoscopic methods for HCR. The colors represent the risk of study bias (green: low, yellow: moderate, red: high). Results are output by the CINeMA website (https://cinema.ispm.unibe.ch/).

**Supplementary Figure 2.**
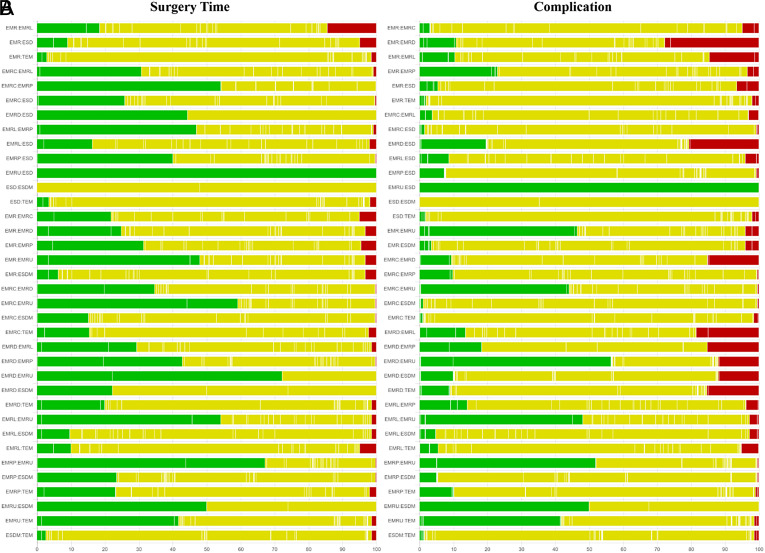
Quality assessment of pairwise comparisons for surgery time and complication. (A) Comparisons on endoscopic methods for surgery time. (B) Comparisons on endoscopic methods for complication. The colors represent the risk of study bias (green: low, yellow: moderate, red: high). Results are output by the CINeMA website (https://cinema.ispm.unibe.ch/).

**Supplementary Figure 3.**
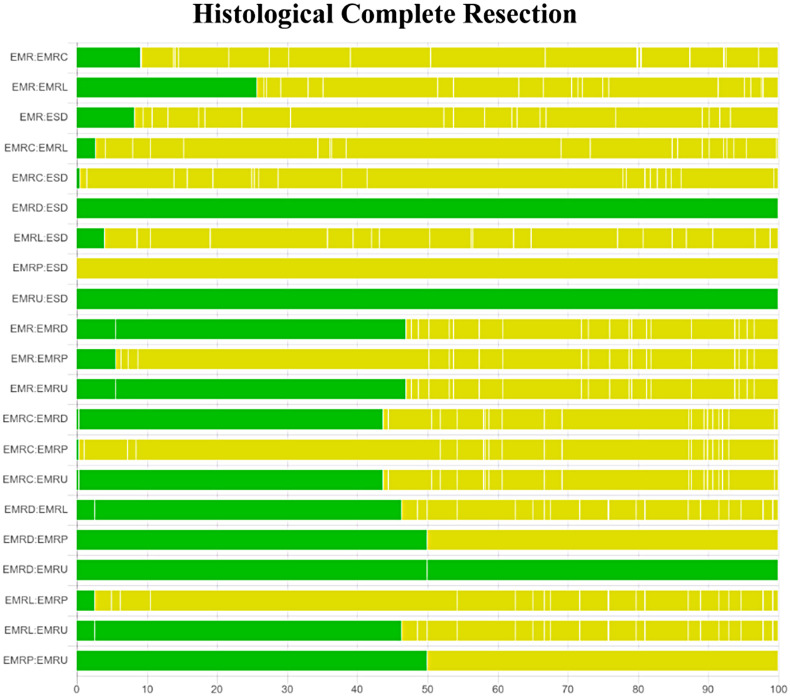
Quality assessment of pairwise comparisons for HCR based on morphology. Comparisons on endoscopic methods for HCR (based on morphology). The colors represent the risk of study bias (green: low, yellow: moderate, red: high). Results are output by the CINeMA website (https://cinema.ispm.unibe.ch/).

**Supplementary Figure 4.**
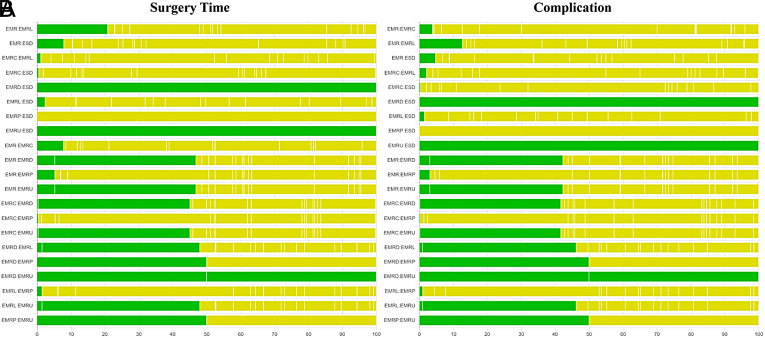
Quality assessment of pairwise comparisons for surgery time and complication based on morphology. (A) Comparisons on endoscopic methods for surgery time (based on morphology). (B) Comparisons on endoscopic methods for complication (based on morphology). The colors represent the risk of study bias (green: low, yellow: moderate, red: high). Results are output by the CINeMA website (https://cinema.ispm.unibe.ch/).

**Supplementary Figure 5.**
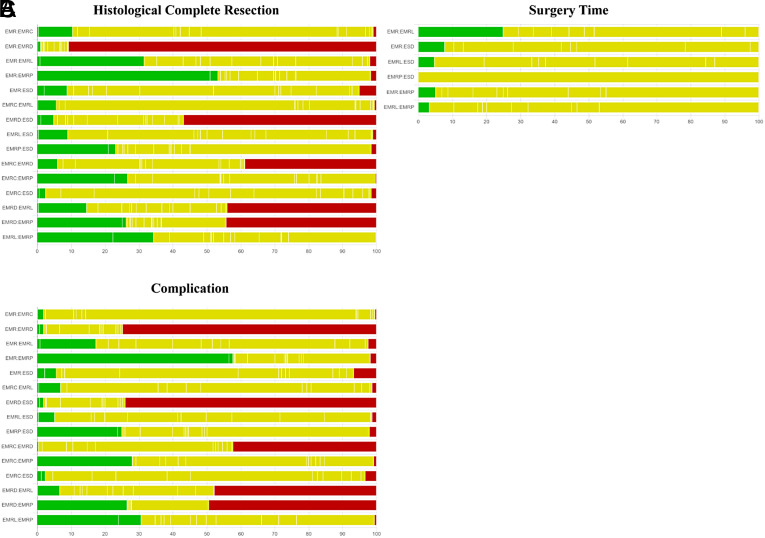
Quality assessment of pairwise comparisons based on histology. (A) Comparisons on endoscopic methods for HCR (based on histology). (B) Comparisons on endoscopic methods for surgery time (based on histology). (C) Comparisons on endoscopic methods for complication (based on histology). The colors represent the risk of study bias (green: low, yellow: moderate, red: high). Results are output by the CINeMA website (https://cinema.ispm.unibe.ch/).

Overall, 1430 literature records were obtained from databases and references (Figure 1). After eliminating duplicate records, screening by reading titles and abstracts, and feasibility of report extraction, 38 retrospective studies^15-52^ were eligible for inclusion in this study, involving 3034 patients (3040 R-NENs). The endoscopic techniques employed in these studies were: EMR with a cap (EMRC), EMR with ligation (EMRL), EMR with pre-cutting (EMRP), EMR with a dual-channel endoscope (EMRD), EMR underwater (EMRU), ESD, ESDM, and TEM. Table 1 presents the characteristics of the 38 eligible studies. Detailed quality assessments of individual studies (Supplementary Table 3) and pairwise comparisons (Supplementary Figures 1- 5) are summarized.

### Network Meta-analysis for Histological Complete Resection

**Figure 2.**
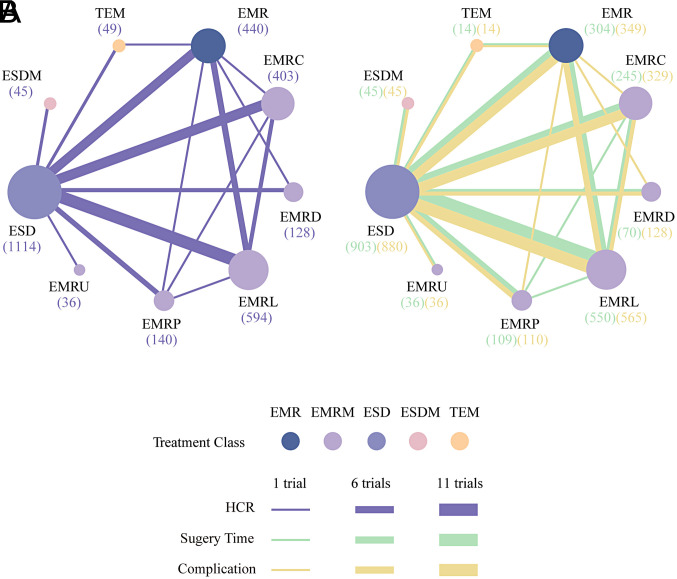
Network diagrams of various comparisons. (A) Comparisons on endoscopic methods for HCR. (B) Comparisons on endoscopic methods for surgery time and complication. Each circular node represents a type of treatment. The node size decreases equally based on the order of sample size receiving treatment (in brackets). Each line represents a type of head-to-head comparison. The width of the lines is proportional to the number of trials comparing the connected treatments. EMR, endoscopic mucosal resection; EMRC, endoscopic mucosal resection with cap; EMRD, endoscopic mucosal resection with dual-channel endoscope; EMRL, endoscopic mucosal resection with ligation; EMRP, endoscopic mucosal resection with pre-cutting; EMRU, endoscopic mucosal resection under water; ESD, endoscopic submucosal dissection; ESDM, modified endoscopic submucosal dissection; HCR, histological complete resection; TEM, transanal endoscopic microsurgery.

**Figure 3.**
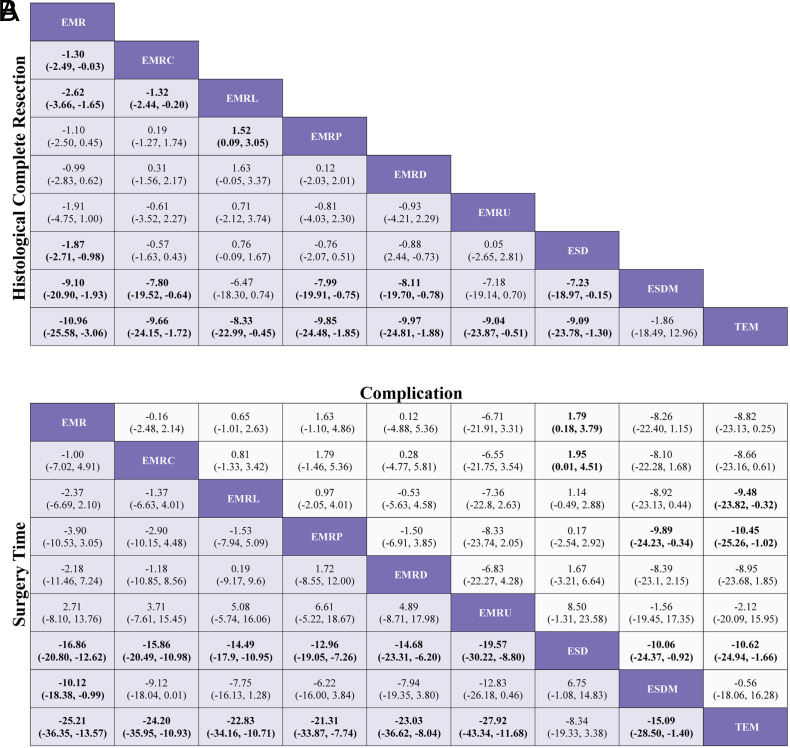
League table of pooled estimates of the network meta-analysis. (A) Log OR (95% CI) for HCR. (B) MD (95% CI) for surgery time (lower triangle) and log OR (95% CI) for complications (upper triangle). Data in each cell are log OR (95% CI) or MD (95% CI) for comparing column-defining treatment versus row-defining treatment. Log OR or MD more than 1 favors column defining treatment. Significant results are in bold. EMR, endoscopic mucosal resection; EMRC, endoscopic mucosal resection with cap; EMRD, endoscopic mucosal resection with dual-channel endoscope; EMRL, endoscopic mucosal resection with ligation; EMRP, endoscopic mucosal resection with pre-cutting; EMRU, endoscopic mucosal resection under water; ESD, endoscopic submucosal dissection; ESDM, modified endoscopic submucosal dissection; HCR, histological complete resection; MD, mean difference; OR, odds ratio; TEM, transanal endoscopic microsurgery.

**Figure 4.**
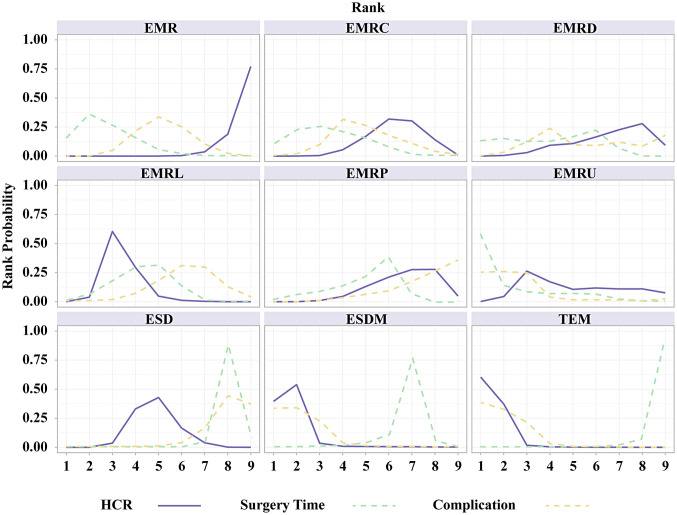
Ranking curves of the network meta-analysis. The figure shows each outcome in different colors. The horizontal axis displays rankings ranging from 1 to 7. The vertical axis shows the probability of being ranked in any specific position, from 0 to 1. EMR, endoscopic mucosal resection; EMRC, endoscopic mucosal resection with cap; EMRD, endoscopic mucosal resection with a dual-channel endoscope; EMRL, endoscopic mucosal resection with ligation; EMRP, endoscopic mucosal resection with pre-cutting; EMRU, endoscopic mucosal resection under water; ESD, endoscopic submucosal dissection; ESDM, modified endoscopic submucosal dissection; TEM, transanal endoscopic microsurgery.

Figures 2A and 3A show the network relationships and effect sizes of the 9 measures for HCR. Using the pairwise comparisons between EMR and EMRMs, EMRC demonstrated a higher capability in achieving HCR than did EMR (log OR, −1.30; 95% CI, −2.49 to −0.03). Endoscopic mucosal resection with ligation showed a higher capability in achieving HCR than did EMRC (−1.32, −2.44 to −0.20) or EMR (−2.62, −3.66 to −1.65). Endoscopic mucosal resection with pre-cutting demonstrated a lower capability in achieving HCR than did EMRL (1.52, 0.09 to 3.05). Endoscopic submucosal dissection demonstrated a higher capability in achieving HCR than did EMR (−1.87, −2.71 to −0.98). Modified endoscopic submucosal dissection demonstrated a higher capability in achieving HCR than did EMR (−9.10, −20.90 to −1.93), EMRC (−7.80, −19.52 to −0.64), EMRP (−7.99, −19.91 to −0.75), EMRD (−8.11, −19.70 to −0.78) and ESD (−7.23, −18.97 to −0.15). Transanal endoscopic microsurgery demonstrated a higher capability in achieving HCR than did EMR (−10.96, −25.59 to −3.06), EMRC (−9.66, −24.15 to −1.72), EMRL (−8.33, −22.99 to −0.45), EMRP (−9.85, −24.48 to −1.85), EMRD (−9.97, −24.81 to −1.88), EMRU (−9.04, −23.87 to −0.51), and ESD (−9.09, −23.78 to −1.30). No statistically significant differences were found in the other comparisons. Figure 4 and Supplementary Table 4 present the ranking of the resectability by these techniques. There were 5 measures that outperformed EMR in achieving HCR. The measure with the highest resectability was probably TEM (SUCRA, 0.95), followed by ESDM (0.91), EMRL (0.70), ESD (0.52), and EMRC (0.34).

### Network Meta-analyses for Surgery Time and Complication

Figures 2B and 3B show the network relationships and effect sizes of the 9 measures of surgery time and complication. Compared to ESD, EMR (MD, −16.86; 95% CI, −20.80 to −12.62), EMRC (−15.86, −20.49 to −10.98), EMRL (−14.49, −17.90 to −10.95), EMRP (−12.96, −19.05 to −7.26), EMRD (−14.68, −23.31 to −6.20), and EMRU (−19.57, −30.22 to −8.80) required a shorter surgery time. Modified endoscopic submucosal dissection was associated with a longer surgery time compared to EMR (−10.12, −18.38 to −0.99). Transanal endoscopic microsurgery was associated with a longer surgery time compared to EMR (−25.21, −36.35 to −13.57), EMRC (−24.20, −35.95 to −10.93), EMRL (−22.83, −34.16 to −10.71), EMRP (−21.31, −33.87 to −7.74), EMRD (−23.03, −36.62 to −8.04), EMRU (−27.92, −43.34 to −11.68), and ESDM (−15.09, −28.50 to −1.40). No statistically significant differences were found in the other comparisons. Figure 4 and Supplementary Table 4 present the ranking of the time-saving of these techniques. There were 6 measures outperforming EMR in regard to time-saving. The measures with the shortest surgery time were probably EMRU (SUCRA, 0.86) and EMR (0.79), followed by EMRC (0.71), EMRL (0.63), EMRP (0.59), and EMRD (0.51). Regarding complication, EMR carried a lower risk than did ESD (log OR, 1.79; 95% CI, 0.18 to 3.79). EMRC carried a lower risk than did ESD (1.95, 0.01 to 4.51). EMRL carried a higher risk compared to TEM (−9.48, −23.82 to −0.32), and EMRP was associated with a higher risk than did ESDM (−9.89, −24.23 to −0.34) or TEM (−10.45, −25.26 to −1.02). ESDM was associated with lower risk than did ESD (10.06, 0.92 to 24.37). TEM carried a lower risk compared to ESD (10.62, 1.66 to 24.94). No statistically significant differences were found in the other comparisons. Figure 4 and Supplementary Table 4 present the ranking of the safety of these techniques. There were 4 measures that outperformed EMR regarding safety. The safest measures probably were TEM (surface under the cumulative ranking curve [SUCRA] 0.87) and ESDM (0.85), followed by EMRC (0.50) and EMR (0.48).

### Subgroup Analyses for Histological Complete Resection, Surgery time, and Complication

**Supplementary Figure 6.**
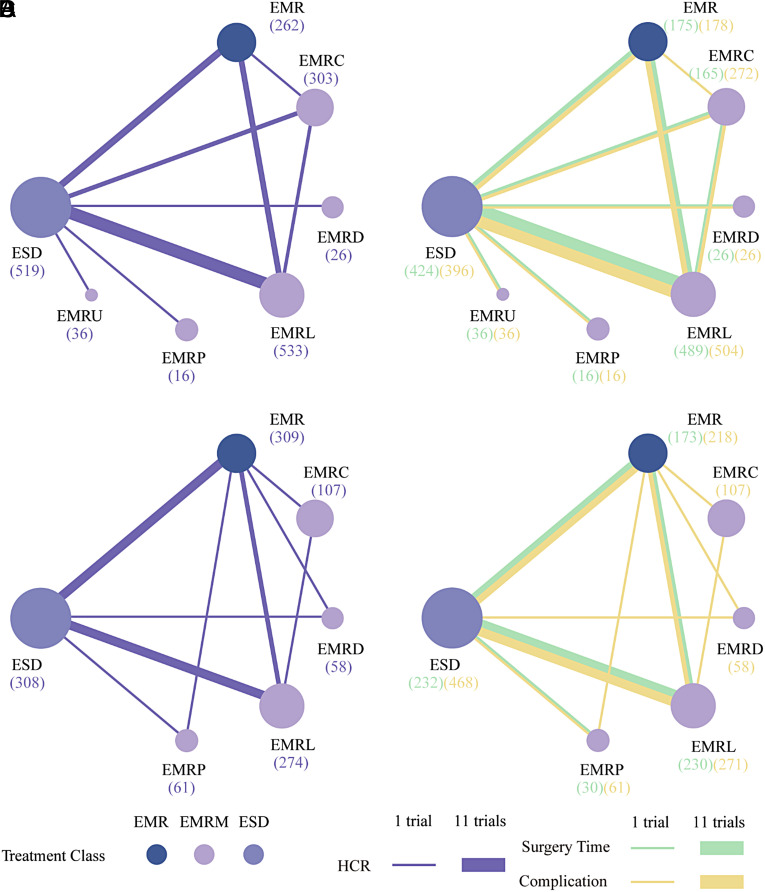
Network diagrams of subgroup analyses. (A) Comparisons on endoscopic methods for HCR (based on morphology). (B) Comparisons on endoscopic methods for surgery time and complication (based on morphology). (C) Comparisons on endoscopic methods for HCR (based on histology). (D) Comparisons on endoscopic methods for surgery time and complication (based on histology). Each circular node represents a type of treatment. The node size decreases by equal disparity in the order of sample size receiving a treatment (in brackets). Each line represents a type of head-to-head comparison. The width of lines is proportional to the number of trials comparing the connected treatments.

**Supplementary Figure 7.**
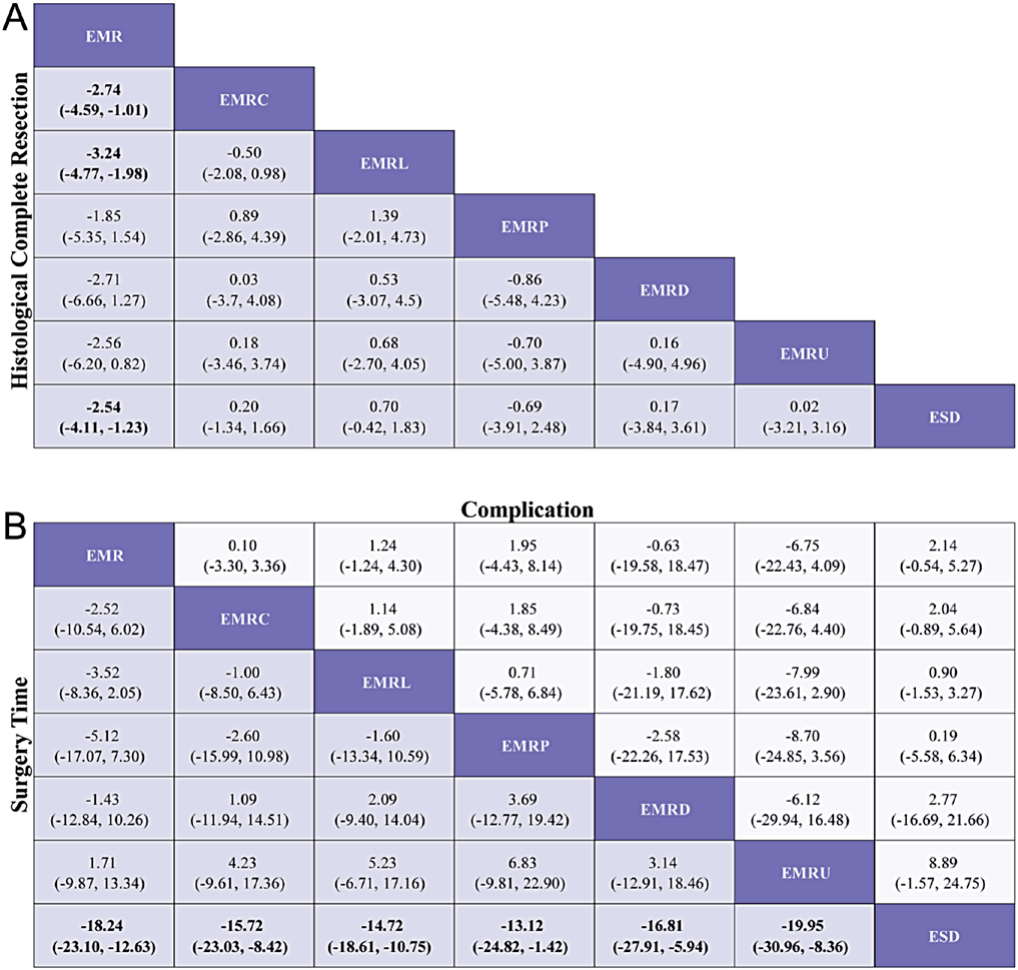
League table of subgroup analysis based on morphology. (A) Log OR (95% CI) for HCR. (B)MD (95% CI) for surgery time lower triangle) and Log OR (95% CI) for complication (upper triangle). Data in each cell are Log OR (95% CI) or MD (95% CI) for the comparison of column-defining treatment versus row- defining treatment. Log OR or MD more than 1 favors column-defining treatment. significant results are in bold.

**Supplementary Figure 8.**
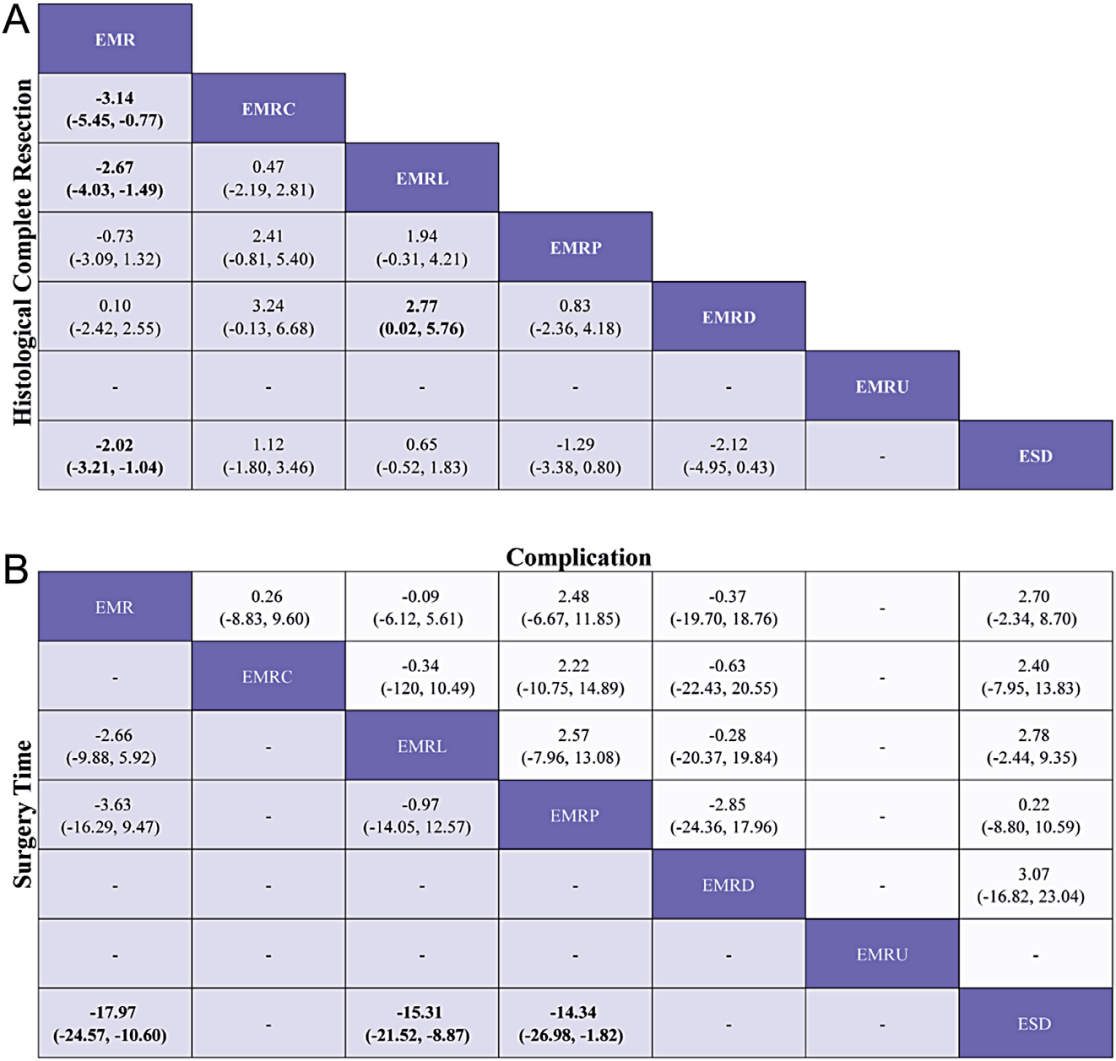
League table of subgroup analysis based on histology. (A) Log OR (95% CI) for HCR. (B)MD (95% CI) for surgery time lower triangle) and Log OR (95% CI) for complication (upper triangle). Data in each cell are Log OR (95% CI) or MD (95% CI) for the comparison of column-defining treatment versus row- defining treatment. Log OR or MD more than 1 favors column-defining treatment. significant results are in bold.

**Supplementary Figure 9.**
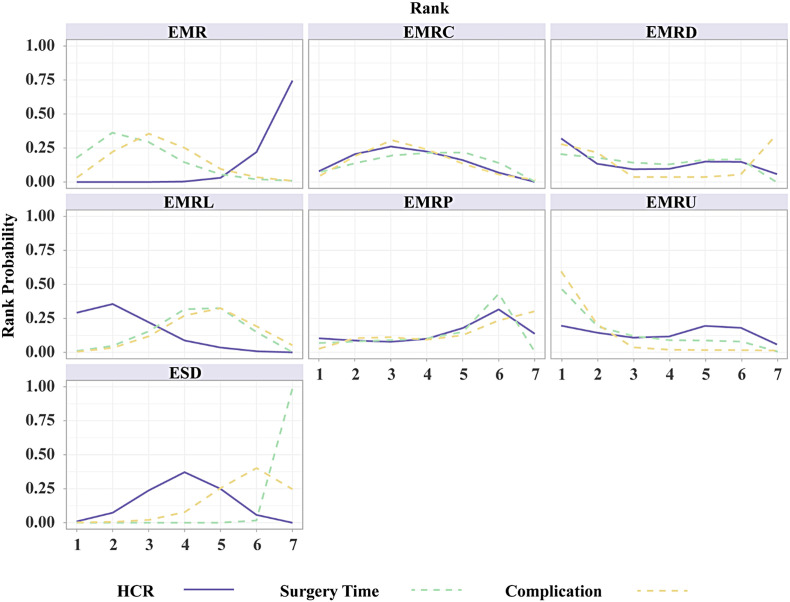
Ranking curves of subgroup analysis based on morphology. The figure shows each outcome with a different color and. The horizontal axis displays the ranking from 1 to 7. The vertical axis displays the probability of being ranked in any specific ranking position, from 0 to 1.

**Supplementary Figure 10.**
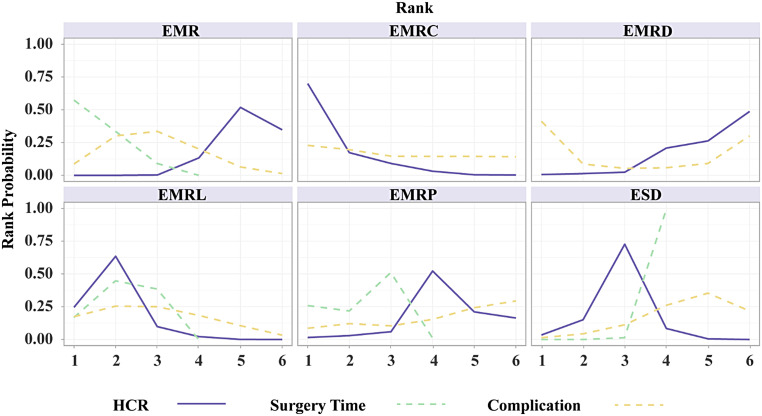
Ranking curves of subgroup analysis based on histology. The figure shows each outcome with a different color and. The horizontal axis displays the ranking from 1 to 7. The vertical axis displays the probability of being ranked in any specific ranking position, from 0 to 1.

The results of subgroup analyses based on morphology and histology, excluding TEM and ESDM, are presented in Supplementary Figures 6-10 and Table 4. In the morphological subgroup, EMRC demonstrated a higher capability in achieving HCR than did EMR (log OR, −2.74; 95% CI, −4.59 to −1.01), EMRL demonstrated a higher capability in achieving HCR than did EMR (−3.24, −4.77 to −1.98), and ESD showed a higher capability in achieving HCR than did EMR (−2.54, −4.11 to −1.23) for HCR. The measures were ranked in the following order: first by EMRL (SUCRA, 0.79), followed by EMRC (0.60), ESD (0.51), and finally by EMR (0.05). Endoscopic mucosal resection (MD, −18.24; 95% CI, −23.10 to −12.63), EMRC (−15.72, −23.03 to −8.42), EMRL (−14.72, −18.61 to −10.75), EMRP (−13.12, −24.82 to −1.42), EMRD (−16.81, −27.91 to −5.94), or EMRU (−19.95, −30.96 to −8.36) took shorter surgery time than did ESD. The measures were ranked in the following order: first by EMRU (SUCRA, 0.77) and EMR (0.74), followed by EMRL (0.60), EMRC (0.54), EMRP (0.44), EMRD (0.41), and finally by ESD (0.00). In the histological subgroup, EMRC demonstrated a higher capability in achieving HCR than did EMR (log OR, −3.14; 95% CI, −5.45 to −0.77). EMRL demonstrated a higher capability in achieving HCR than did EMR (−2.67, −4.03 to −1.49). In addition, ESD demonstrated a higher capability in achieving HCR than did EMR (−2.02, −3.21 to −1.04) for HCR. The techniques were ranked in the following order: first by EMRC (SUCRA, 0.91), followed by EMRL (0.82), ESD (0.63), and finally by EMR (0.16). EMR (MD, −17.97; 95% CI, −24.57 to −10.60), EMRL (−15.31, −21.52 to −8.87), EMRP (−14.34, −26.98 to −1.82) took shorter than did ESD for surgery time. The techniques were ranked in the following order: first by EMR (SUCRA, 0.82), followed by EMRL (0.61), EMRP (0.56), and finally by ESD (0.01). In addition, pairwise comparisons across the 7 and 6 measures in the 2 subgroups revealed similar risks of complication.

### Heterogeneity, Inconsistency, and Reporting Bias

**Supplementary Figure 11.**
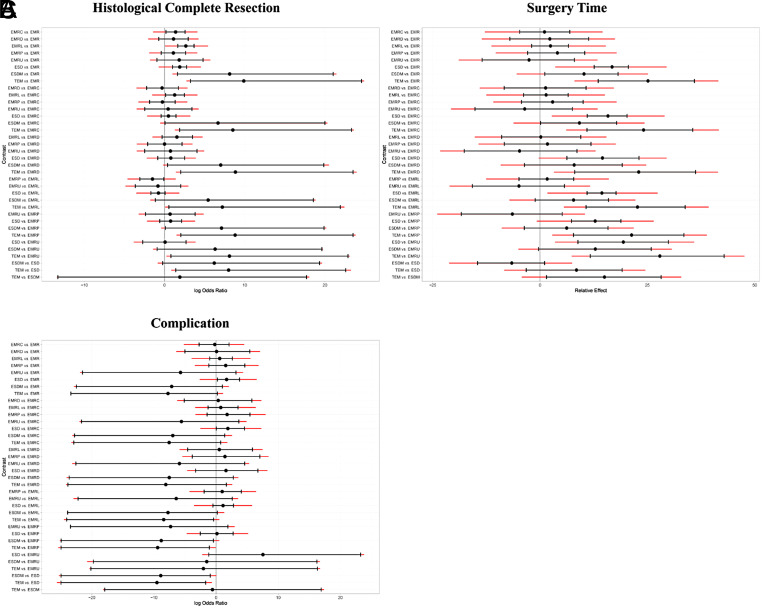
Forest plot of network meta-analysis between all measures. (A) Comparisons on endoscopic methods for HCR. (B) Comparisons on endoscopic methods for surgery time. (C) Comparisons on endoscopic methods for complication. The horizontal ordinates of black nodes represent the effect sizes (log ORs or MDs) for each pairwise comparison, the black lines represent the 95% CIs and the red lines represent the 95% PrI. No imprecision is considered to exist when the black lines do not cross the vertical axis and no local heterogeneity is considered to exist when both the red and black lines synchronously cross or synchronously uncross the vertical axis.

**Supplementary Figure 12.**
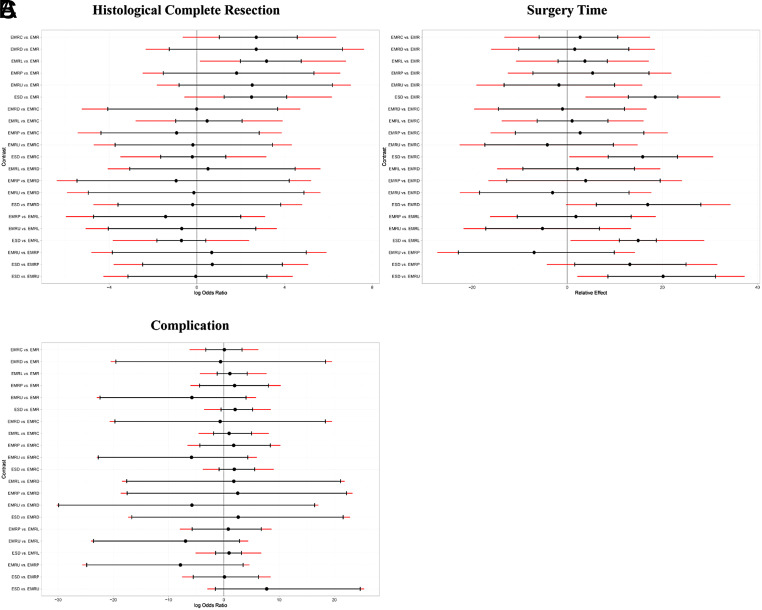
Forest plot of subgroup analysis based on morphology. (A) Comparisons on endoscopic methods for HCR. (B) Comparisons on endoscopic methods for surgery time. (C) Comparisons on endoscopic methods for complication. The horizontal ordinates of black nodes represent the effect sizes (log ORs or MDs) for each pairwise comparison, the black lines represent the 95% CIs and the red lines represent the 95% PrI. No imprecision is considered to exist when the black lines do not cross the vertical axis and no local heterogeneity is considered to exist when both the red and black lines synchronously cross or synchronously uncross the vertical axis.

**Supplementary Figure 13.**
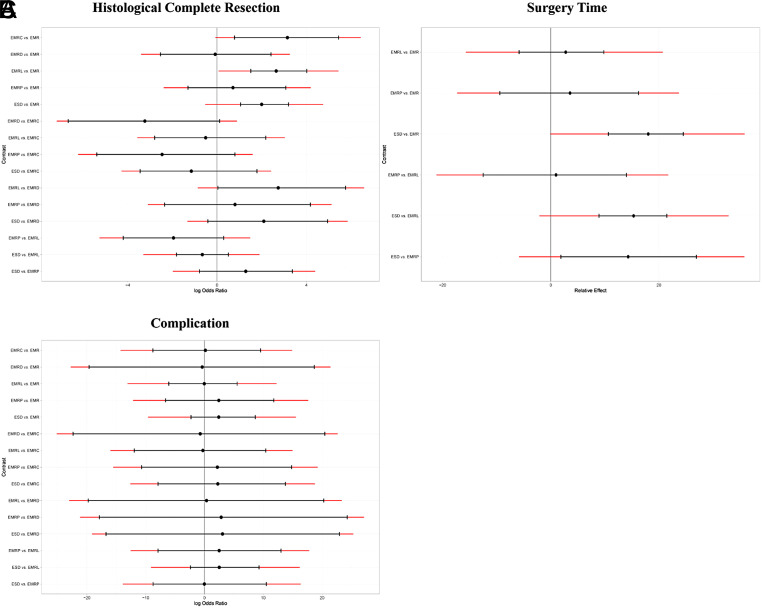
Forest plot of subgroup analysis based on histology. (A) Comparisons on endoscopic methods for HCR. (B) Comparisons on endoscopic methods for surgery time. (C) Comparisons on endoscopic methods for complication. The horizontal ordinates of black nodes represent the effect sizes (log ORs or MDs) for each pairwise comparison, the black lines represent the 95% CIs and the red lines represent the 95% PrI. No imprecision is considered to exist when the black lines do not cross the vertical axis and no local heterogeneity is considered to exist when both the red and black lines synchronously cross or synchronously uncross the vertical axis.

**Supplementary Figure 14.**
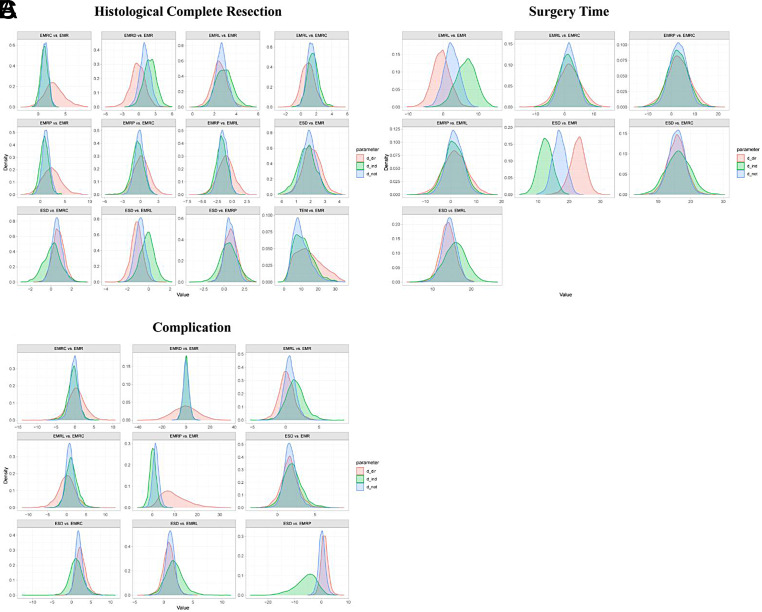
Node-splitting plot of network meta-analysis between all measures. (A) Comparisons on endoscopic methods for HCR. (B) Comparisons on endoscopic methods for surgery time. (C) Comparisons on endoscopic methods for complication. The red arc area represents the direct effect, the green arc area represents the indirect effect, and the blue arc area represents the mixed effect. The extent of overlap between the three represents local inconsistency, the higher the extent of overlap, the less significant the local inconsistency, and vice versa, the significant the local inconsistency.

**Supplementary Figure 15.**
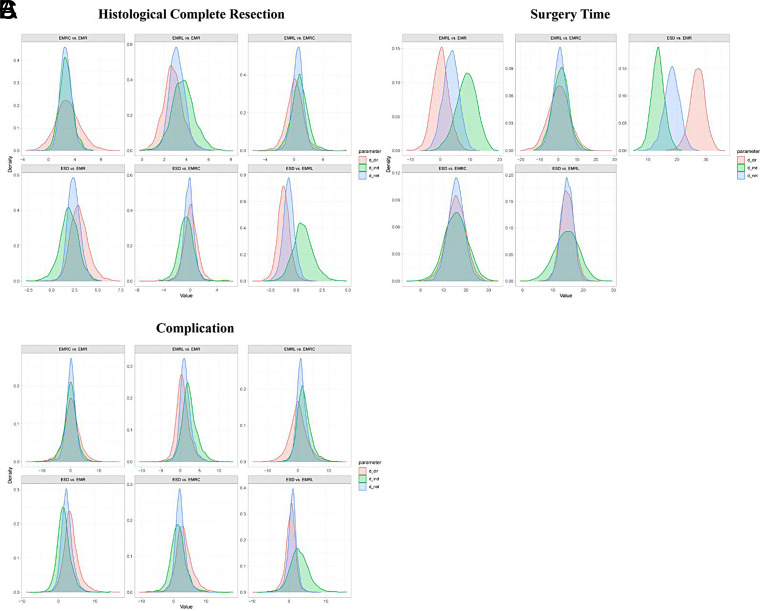
Node-splitting plot of subgroup analysis based on morphology. (A) Comparisons on endoscopic methods for HCR. (B) Comparisons on endoscopic methods for surgery time. (C) Comparisons on endoscopic methods for complication. The red arc area represents the direct effect, the green arc area represents the indirect effect, and the blue arc area represents the mixed effect. The extent of overlap between the three represents local inconsistency, the higher the extent of overlap, the less significant the local inconsistency, and vice versa, the significant the local inconsistency.

**Supplementary Figure 16.**
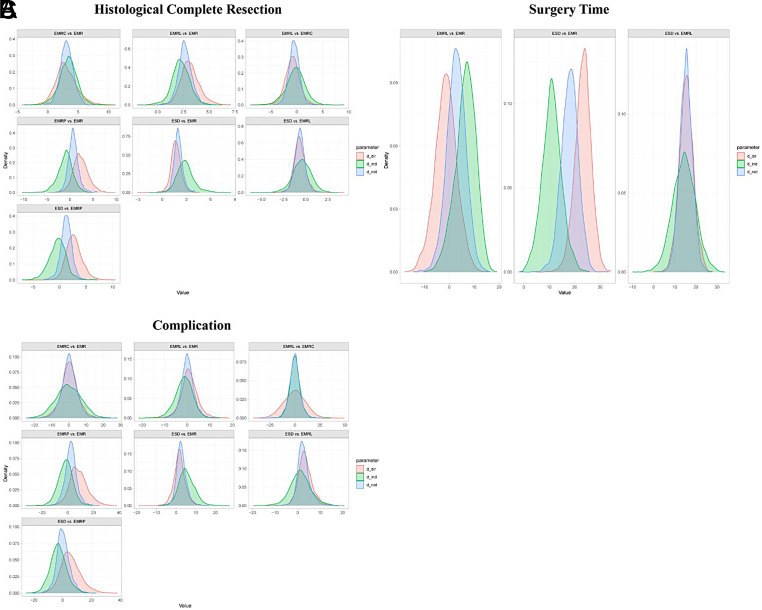
Node-splitting plot of subgroup analysis based on histology. (A) Comparisons on endoscopic methods for HCR. (B) Comparisons on endoscopic methods for surgery time. (C) Comparisons on endoscopic methods for complication. The red arc area represents the direct effect, the green arc area represents the indirect effect, and the blue arc area represents the mixed effect. The extent of overlap between the three represents local inconsistency, the higher the extent of overlap, the less significant the local inconsistency, and vice versa, the significant the local inconsistency.

**Supplementary Figure 17.**
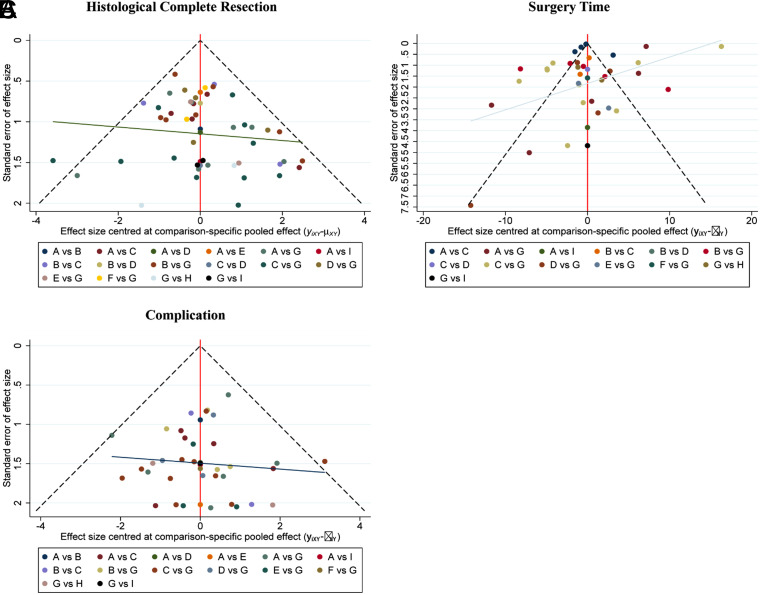
Funnel plot of network meta-analysis between all measures. (A) Comparisons on endoscopic methods for HCR. (B) Comparisons on endoscopic methods for surgery time. (C) Comparisons on endoscopic methods for complication. The dots represent the original study and are considered to have no significant publication bias when they appear visually symmetrical and almost all the dots fall within the triangular area formed by the dashed line and the horizontal axis, otherwise the publication bias is considered significant.

**Supplementary Figure 18.**
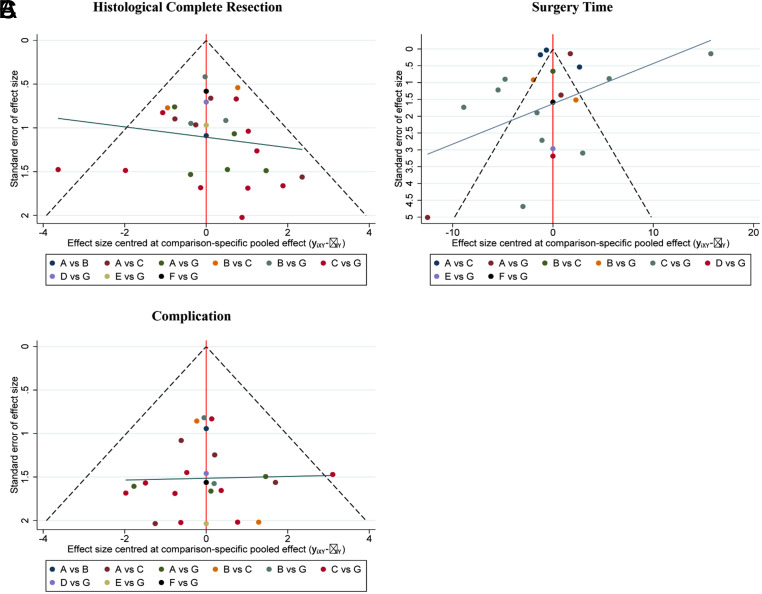
Funnel plot of subgroup analysis based on morphology. (A) Comparisons on endoscopic methods for HCR. (B) Comparisons on endoscopic methods for surgery time. (C) Comparisons on endoscopic methods for complication. The dots represent the original study and are considered to have no significant publication bias when they appear visually symmetrical and almost all the dots fall within the triangular area formed by the dashed line and the horizontal axis, otherwise the publication bias is considered significant.

**Supplementary Figure 19.**
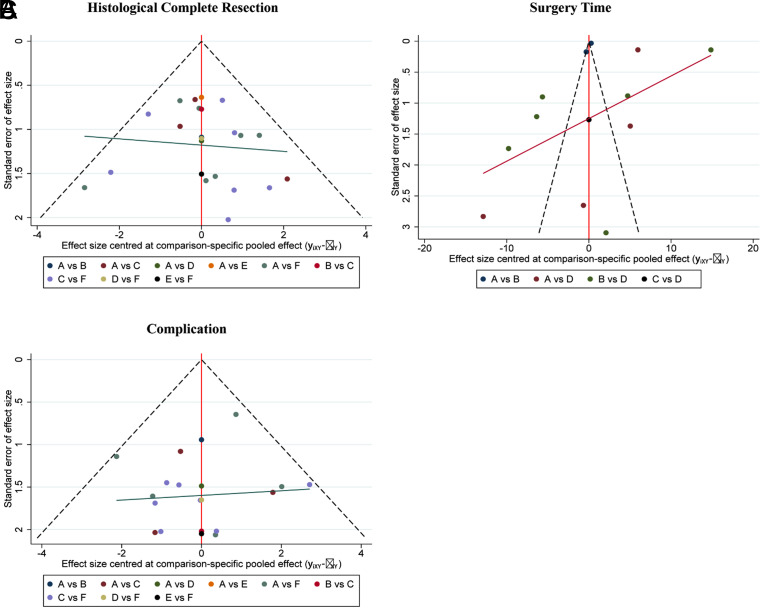
Funnel plot of subgroup analysis based on histology. (A) Comparisons on endoscopic methods for HCR. (B) Comparisons on endoscopic methods for surgery time. (C) Comparisons on endoscopic methods for complication. The dots represent the original study and are considered to have no significant publication bias when they appear visually symmetrical and almost all the dots fall within the triangular area formed by the dashed line and the horizontal axis, otherwise the publication bias is considered significant.

There was no evidence of statistically significant global heterogeneity or global inconsistency regarding HCR, surgery time, or complication (Supplementary Table 5 and 6). However, partial local heterogeneity and inconsistency were found (Supplementary Figures 11-16 and Table 6). No significant reporting bias was found in HCR or complication. However, the main and subgroup analyses indicated significant bias in surgery time (Supplementary Figures 17-19).

### Network Regression Analyses

Network regression was performed with en bloc resection rate, clarity of surgery time, clarity of complication, publication year to evaluate the effect of definition differences on outcomes, with patient’s age, sex, and tumor location (distance from the anal verge) to evaluate the effect of patient and tumor conditions on outcomes. The influence of the above factors was not found (Supplementary Table 7).

### Assessment of Evidence Certainty

Network meta-analysis included 74 mixed, 17 direct, and 116 indirect comparisons. In the GRADE assessment, 12, 132, and 63 comparisons were judged to have moderate, low, and very low certainty evidence, respectively (Supplementary Table 8).

## Discussion

Most rectal NENs manifest no carcinoid syndrome or typical clinical symptoms and are often discovered incidentally during routine colonoscopy. Therefore, with the popularization of colonoscopy, the incidence of rectal NENs is increasing every year.^2^ Endoscopic treatment is currently the recommended modality for R-NENs with low malignant potential, including EMR, EMRM, ESD, ESDM, EFR, and TEM.^5^

Recently, EMR is no longer used for treating R-NENs ≤20 mm due to its weak resectability. Endoscopic submucosal dissection requires prior delineation of a circumferential area around the lesion with an electrocauterization knife to enable submucosal resection to be performed under direct visualization for achieving a deeper and wider resection. Due to its high integrity and low risk of residue and recurrence, ESD is considered the standard excision technique for early-stage gastrointestinal tract cancers.^53^ This explains the results of a previous meta-analysis^7,54^ and our NMA. Due to the large resection range of ESD, procedure-related bleeding and perforation were usually more likely to occur compared to EMR.^55^ However, there is still controversy over the risk of complications for smaller R-NENs. Yong et al^7^ suggested that the bleeding risk of ESD for R-NENs between 10-20 mm was concerning, whereas the bleeding risk of ESD for R-NENs ≤10mm was acceptable as EMR. Zhou et al^54^ revealed that for R-NENs ≤15mm with low malignant potential, the risk of complications of ESD was similar to that of EMR. Regarding R-NENs ≤20 mm with endoscopically suspected low malignant potential, the risk of complications between ESD and EMR remains uncertain. Considering R-NENs ≤20 mm with pathologically confirmed low malignant potential or R-NENs ≤10 mm with endoscopically suspected low malignant potential, our study indicated that the risk of complications was similar between ESD and EMR. These results emphasized the importance of improving the accuracy of preoperative diagnosis of R-NENs. ESD was often associated with higher medical expenditures compared to EMR due to higher treatment costs and longer hospital stays. Although it was not possible to analyze the cost-effectiveness of ESD due to the significant differences in healthcare costs between countries and regions, confirmed by several studies^16,29^ and clinical realities. In addition, popularizing ESD due to operational difficulty and instrument requirement is still difficult.

To overcome the limitations of EMR and ESD, various EMRMs have been developed. However, the most suitable technique for treating R-NENs with low malignant potential remains unknown. In earlier meta-analyses, EMRMs were usually compared to EMR or ESD, considered as a whole.^6,54^ However, there was no comprehensive comparison between each EMRM. This makes it difficult for inexperienced endoscopists to make sensible decisions in practice because there are still variations in their methodology and application. Therefore, we derived different recommendation levels for different techniques by comparing each EMRM to EMR and ESD separately and analyzing them with the effect rankings.

EMRL uses ligation-assisted instruments, such as bands or clips, to sufficiently lift the tumor tissue, allowing for resecting lesions deeper in the submucosa compared to conventional EMR.^41^ This is consistent with our findings. Due to the ongoing controversy about EMRMs over the treatment of R-NENs between 10-20 mm,^56^ the maximum tumor diameter in the original studies on EMRL in this NMA is only up to 16 mm. Therefore, we suggest that EMRL might outperform EMR regarding resectability of R-NENs ≤16 mm with low malignant potential. In addition, Lim et al.^38^ indicated that EMRL had a wider resection range than did ESD to achieve a higher HCR rate for R-NENs ≤10 mm. Although the difference between EMRL and ESD is not statistically significant, EMRL ranks higher in HCR rate than does ESD. EMRC uses negative pressure to aspirate an elevated lesion into a transparent cap before resecting it, providing similar resectability and safety to ESD but with a shorter duration.^37^ However, due to the transparent cap’s poor freedom and limited volume, EMRC may be more suitable for smaller tumor compared to ESD. Consistent with most studies,^27,41^ our study revealed that EMRC could be more suitable for the treatment of R-NENs ≤10mm. When tumor size is appropriate, EMRC can even achieve a resection effect that is not inferior to EMRL. EMRP involves injecting saline into the submucosa using an injection needle to augment the lesion and create a circumferential incision (pre-incision) using the tip of a loop or special endoscopic cutter, and removing the tumor with the loop. The advantage of EMRP over other EMRMs is that there is no limitation on the size of the resected tumor.^26^ EMRD uses a dual-channel endoscope to lift the lesion with forceps and snare it.^15^ However, dual-channel endoscopy is not widely used. EMRU achieves deeper lesion resection by filling the intestinal cavity with water.^43^ Although studies have shown that EMRP, EMRD and EMRU could replace ESD for treating R-NENs ≤10mm or 15mm,^22,28^ we found that the therapeutic benefit of them was not superior to that of EMR. Therefore, caution should be exercised when selecting EMRP, EMRC, or EMRU. Regarding surgery time, EMRL and EMRC might cost less compared to ESD for R-NENs ≤16mm with low malignant potential, while EMRU might cost less compared to ESD for R-NENs ≤10mm with low malignant potential. Regarding complication risk, we considered that each EMRMs might be relatively similar to EMR.

This study included 2 modified ESD procedures: C-type ESD^52^ (replacing the pre-delineated circumferential area with a C-shaped area) and dental floss traction-assisted ESD.^39^ Our results revealed that these modified techniques could improve the performance of ESD in terms of both resectability and safety. ESDMs would be good endoscopic treatments if were technically feasible due to their better therapeutic efficacy and similar safety compared to EMRMs. Although the quality of evidence suggesting that the insignificant difference between ESDM and ESD in surgery time was very low, ESDM is a modified technique of ESD. Therefore, we suggest that the difference in surgery time between them should not be significant, which is consistent with the views of the studies we included.^39,52^ TEM, which combines the advantages of traditional transanal rectal surgery and laparoscopic surgery, can be easily used for resecting R-NENs and salvage treatment.^24^ Our findings indicated that TEM likely outperformed ESD in terms of resectability and safety for R-NENs ≤20 mm with endoscopically suspected low malignant potential. However, since TEM is more technically and equipment-demanding compared to ESD, resulting in longer procedure times and hospital stays and higher medical expenditures,^57^ the endoscopists should pay careful attention to the above issues.

This study has several limitations: First, because there are too few prospective randomized studies comparing endoscopic techniques, the inherent selection bias of retrospective studies included in this NMA might be inevitable. Second, although we conducted subgroup analysis and regression analysis of various confounding factors, there are still some significant local heterogeneity and inconsistency, which may be due to the inherent limitations of methodology of NMA. Therefore, we critically assessed the quality of evidence, which readers may apply with caution given the results and quality of evidence. Third, since R-NENs are prevalent in Asian populations, most of the published articles now originate from Asia,^2^ and the analysis based on this data may have limited generalizability to other populations. Last, the present study is limited to exploring the surgery time and medical cost for various endoscopic technique due to the significant differences in different countries or region. In conclusion, more high-quality randomized controlled studies need to be conducted in to address these limitations.

Regarding R-NENs ≤20 mm with low malignant potential, ESD could outperform EMR in terms of resectability, whereas safety remains a concern. Transanal endoscopic microsurgery could outperform ESD in terms of resectability and safety, whereas surgery time and medical cost were concerns. Endoscopic submucosal dissection can be used as the primary treatment. However, TEM may be more effective if supported by economic conditions and hospital facility. In regard to R-NENs ≤16 mm with low malignant potential, EMRL was found to combine the resectability by ESD with the safety of EMR with shorter operative time and lower cost compared to ESD. Endoscopic mucosal resection with ligation could be used as the primary treatment. With respect to R-NENs ≤10 mm with low malignant potential, EMRL, EMRC and ESD could have better resectability and similar safety compared to EMR, whereas EMRL and EMRC showed also shorter time and lower cost compared to ESD. Endoscopic mucosal resection with ligation, EMRC and ESD could be used as the primary treatment. However, EMRL and EMRC might be better when taking into account the operational difficulties and economic conditions.

## Figures and Tables

**Table 1.** Baseline Characteristics of Studies Included in the Network Meta-Analysis

Author, Year	Country	Group Comparisons	Patients’ Feature			Endoscopic Feature			Pathological Feature		
			No. of Patients	No. of Lesions	Age, Years (Mean ± SD)	Male (%)	Size, mm (Range)	Size, mm (Mean ± SD)	Size, mm (Mean ± SD)	G1-G2 (%)	Lymphovascular Invasion (%)
Onozato et al^15^	Japan	A: EMRD	24	26	NA	75.0	≤10 mm	NA	6.6 ± 2.1	NA	0
		B: ESD	9	9	NA	77.8		NA	7.7 ± 1.0	NA	0
Zhou et al^16^	China	A: EMR	23	23	50.3 ± 13.6	60.9	≤10 mm	6.7 ± 2.1	NA	NA	NA
		B: ESD	20	20	47.6 ± 18.5	60.0		7.2 ± 1.9	NA	NA	NA
Sung et al^17^	Korea	A: EMR	14	14	NA	NA	<15 mm	NA	NA	100	0
		B: EMRD	58	58	NA	NA		NA	NA	100	0
		C: ESD	5	5	NA	NA		NA	NA	100	0
Kim et al^18^	Korea	A: EMRL	45	45	53.5 ± 10.4	68.9	≤10 mm	5.9 ± 2.0	5.8 ± 2.4	100	0
		B: EMR	55	55	48.8 ± 13.9	63.6		6.3 ± 2.5	6.5 ± 3.2	100	0
Niimi et al^19^	Japan	A: ESD	13	13	55.3 ± 8.6	69.2	≤10 mm	5.4 ± 1.4	5.5 ± 2.1	NA	NA
		B: EMRL	11	11	45.5 ± 10.6	72.7		5.7 ± 2.1	4.4 ± 2.2	NA	NA
Heo et al^20^	Korea	A: EMRL	48	48	49.7 ± 12.5	52.1	<10 mm	5.9 ± 1.9	5.3 ± 2.6	100	2.0
		B: EMR	34	34	49.9 ± 10.8	73.5		6.2 ± 2.9	7.0 ± 2.8	100	0
Choi et al^21^	Korea	A: EMRL	29	29	47.8 ± 11.7	51.7	<10 mm	4.3 ± 1.8	NA	100	0
		B: ESD	31	31	48.3 ± 14.4	64.5		5.2 ± 2.1	NA	100	0
Dou et al^22^	China	A: EMR	24	24	49.0 ± 8.3	62.5	<20 mm	5.6 ± 1.2	NA	100	0
		B: ESD	19	19	48.6 ± 9.0	52.6		7.4 ± 5.3	NA	100	0
Lee et al^23^	Korea	A: EMRD	44	44	51.4 ± 12.3	56.8	<16 mm	6.4 ± 2.7	NA	NA	NA
		B: ESD	26	26	47.4 ± 10.6	88.5		6.2 ± 4.1	NA	NA	NA
Jeon et al^24^	Korea	A: EMR	29	29	47.6 ± 9.6	79.3	<20 mm	6.1 ± 2.3	NA	100	0
		B: ESD	23	23	51.0 ± 12.3	65.2		6.7 ± 1.8	NA	100	0
		C: TEM	14	14	8.5 ± 14.4	64.3		8.2 ± 3.0	NA	100	7.0
Im et al^25^	Korea	A: EMRL	35	35	49.4 ± 9.2	57.1	<15 mm	6.1 ± 2.1	NA	NA	0
		B: ESD	74	74	51.9 ± 9.9	64.9		6.1 ± 2.8	NA	NA	1.0
Huang et al^26^	China	A: EMRP	31	31	50 ± 10.1	54.8	<15 mm	NA	9.0 ± 2.5	100	0
		B: EMR	28	28	49.0 ± 12.2	53.6		NA	8.0 ± 3.3	100	0
Wang et al^27^	China	A: EMRC	30	30	45.2 ± 6.2	56.7	7-16 mm	10.4 ± 3.0	NA	NA	0
		B: ESD	25	25	44.5 ± 6.0	52.0		12.3 ± 2.8	NA	NA	0
Li^28^	China	A: EMR	35	35	51.5 ± 10.9	62.9	<10 mm	5.0 ± 2.0	NA	100	0
		B: EMRC	42	42	52.6 ± 11.8	52.4		6.0 ± 2.0	NA	100	0
Cheung et al^29^	Korea	A: ESD	17	17	46.3 ± 8.6	64.7	≤10 mm	7.5 ± 1.9	NA	NA	0
		B: EMRP	16	16	51.5 ± 8.8	93.8		6.6 ± 2.0	NA	NA	0
Chen et al^30^	China	A: EMRP	33	33	51.6 ± 8.5	51.5	<15 mm	6.9 ± 2.9	NA	NA	0
		B: ESD	28	28	50.9 ± 9.83	60.7		8.2 ± 2.9	NA	NA	0
Yang et al^31^	China	A: EMRL	27	27	49.8 ± 8.4	55.6	≤10 mm	6.3 ± 1.4	NA	NA	NA
		B: ESD	19	19	50.2 ± 8.6	57.9		6.2 ± 1.4	NA	NA	NA
Bang et al^33^	Korea	A: EMRL	53	53	53.6 ± 12.7	60.4	≤10 mm	5.0 ± 1.7	4.6 ± 1.7	100	1.9
		B: ESD	24	24	50.8 ± 12.4	75.0		5.5 ± 2.1	5.2 ± 1.9	100	8.3
Choi et al^32^	Korea	A: EMRC	65	65	NA	67.7	≤10mm	NA	NA	100	0
		B: EMRL	16	16	NA	68.8		NA	NA	100	0
		C: ESD	53	53	NA	60.4		NA	NA	100	1.9
Zhang et al^34^	China	A: EMRP	30	30	49.6 ± 12.4	53.3	≤16 mm	6.9 ± 2.5	NA	100	0
		B: ESD	36	36	45.5 ± 12.2	44.4		7.2 ± 2.4	NA	100	0
Zhang et al^35^	China	A: EMRC	29	29	45.4 ± 13.1	55.2	<10 mm	6.3 ± 1.6	NA	NA	0
		B: ESD	23	23	46.8 ± 14.2	56.5		6.4 ± 1.7	NA	NA	0
Yang et al^36^	China	A: EMRC	27	27	48.0 ± 6.8	55.6	<13 mm	5.1 ± 1.2	NA	100	0
		B: ESD	15	15	51.0 ± 7.5	60.0		6.8 ± 2.1	NA	100	6.7
Wang et al^37^	China	A: EMR	22	22	57.6 ±9.8	59.1	<10 mm	6.4 ± 1.5	NA	100	0
		B: EMRL	20	20	54.3 ± 12.3	65.0		6.0 ± 1.1	NA	100	0
		C: ESD	7	7	60.3 ± 12.6	85.7		6.9 ± 1.2	NA	100	0
Lim et al^38^	Korea	A: EMRL	66	66	51.6 ± 9.8	56.1	<10 mm	5.0 ± 1.7	NA	100	0
		B: ESD	16	16	52.7 ± 9.8	50.0		7.1 ± 2.2	NA	100	0
Shi et al^39^	China	A: ESDM	17	17	58.0 ± 9.3	58.8	<15 mm	8.0 ± 2.0	NA	100	NA
		B: ESD	20	20	54.3 ± 12.8	65.0		7.0 ± 2.0	NA	100	NA
Wu et al^40^	China	A: EMRL	23	23	49.8 ± 8.4	56.5	<10 mm	6.8 ± 1.6	NA	100	0
		B: ESD	20	20	52.2 ± 7.6	55.0		7.3 ± 1.1	NA	100	0
Lee et al^41^	Korea	A: EMRC	158	42	49.2 ± 12.2	54.8	<10 mm	4.6 ± 2.3	NA	100	2.4
		B: EMRL		120	51.7 ± 10.7	56.7		4.8 ± 1.9	NA	100	0.8
Wang et al^42^	China	A: EMRC	23	23	51.4 ± 12.6	52.2	<20 mm	6.1 ± 1.8	NA	100	NA
		B: EMRL	26	26	48.6 ± 8.6	73.1		5.4 ± 2.0	NA	100	NA
		C: EMRP	30	30	51.2 ± 11.9	53.3		6.0 ± 1.9	NA	100	NA
		D: ESD	259	259	49.2 ± 10.7	61.4		7.1 ± 3.1	NA	100	NA
Park et al^43^	Korea	A: EMRU	36	36	45.7 ± 12.2	61.1	≤10 mm	5.0 ± 2.0	NA	100	NA
		B: ESD	79	79	47 ± 10.3	63.3		5.0 ± 2.0	NA	100	NA
Wang et al^44^	China	A: ESD	28	28	47.8 ± 10.0	60.7	<10 mm	6.8 ± 2.5	NA	100	0
		B: EMR	41	41	52.8 ± 11.1	70.7		6.7 ± 2.3	NA	100	0
Liang^45^	China	A: EMRC	94	94	43.5 ± 11.8	63.8	≤10 mm	6.6 ± 1.9	NA	100	NA
		B: ESD	67	67	45.2 ± 12.1	70.1		7.1 ± 2.1	NA	100	NA
Chen and Liang^46^	China	A: EMRC	31	31	51.03 ± 10.16	51.6	<7 mm	4.9 ± 0.9	NA	NA	NA
		B: ESD	34	34	49.8 ± 13.5	64.7		5.2 ± 0.8	NA	NA	NA
Chen and Liang^46^	China	A: EMRC	20	20	48.9 ± 8.5	45.0	7-15 mm	9.2 ± 2.0	NA	NA	NA
		B: ESD	25	25	48.2 ± 10.3	64.0		9.0 ± 1.7	NA	NA	NA
Wang et al^47^	China	A: ESD	76	76	50.3 ± 12.5	47.4	≤20 mm	NA	NA	100	2.6
		B: TEM	35	35	53.8 ± 12.2	48.6		NA	NA	100	2.9
Li et al^48^	China	A: ESD	21	21	55.3 ± 11.6	47.6	≤10 mm	6.8 ± 2.6	NA	100	0
		B: EMRL	21	21	54.4 ± 10.6	42.9		5.8 ± 2.2	NA	100	0
Hu^49^	China	A: EMR	50	50	NA	NA	≤20 mm	7.0 ± 3.2	NA	100	0
		B: ESD	40	40	NA	NA		7.6 ± 3.5	NA	100	0
Jiang^50^	China	A: EMRL	28	28	NA	35.7	≤10 mm	6.6 ± 2.0	NA	100	0
		B: ESD	31	31	NA	61.3		6.1 ± 2.0	NA	100	0
		C: EMR	9	9	NA	33.3		6.0 ± 1.9	NA	100	0
Chen et al^51^	China	A: EMRL	26	26	49.0 ± 11.7	46.2	<10 mm	7.1 ± 2.8	NA	100	0
		B: ESD	30	30	52.8 ± 10.9	60.0		7.5 ± 2.3	NA	100	0
Wu et al^52^	China	A: ESDM	28	28	48.1 ± 11.2	57.1	<15 mm	4.0 (3.0–6.0)^2^	NA	100	NA
		B: ESD	27	27	46.2 ± 9.1	55.6		5.0 (4.0–5.0)^2^	NA	100	NA

EMR, endoscopic mucosal resection; EMRC, endoscopic mucosal resection with cap; EMRD, endoscopic mucosal resection with dual-channel endoscope; EMRL, endoscopic mucosal resection with ligation; EMRP, endoscopic mucosal resection with pre-cutting; EMRU, endoscopic mucosal resection under water; ESD, endoscopic submucosal dissection; ESDM, modified endoscopic submucosal dissection; NA, not available; TEM, transanal endoscopic microsurgery.

^1^Two subgroups in the same study.

^2^Data are represented by median (Q1-Q3).

**Supplementary Table 1.** Checklist of the PRISMA Extension for Network Meta-analysis

**Section/topic**			**Pages**
**TITLE**			
Title	1	Identify the report as a systematic review incorporating a network meta-analysis (or related form of meta-analysis).	
**ABSTRACT**			
Structured summary	2	Provide a structured summary including, as applicable: Background: main objectives; Methods: data sources; study eligibility criteria, participants, and interventions; study appraisal and synthesis methods, such as network meta-analysis. Results: number of studies and participants identified; summary estimates with corresponding confidence/credible intervals; treatment rankings may also be discussed. Authors may choose to summarize pairwise comparisons against a chosen treatment included in their analyses for brevity. Discussion/Conclusions: limitations; conclusions and implications of findings. Other: primary source of funding; systematic review registration number with registry name.	
**INTRODUCTION**			
Rationale	3	Describe the rationale for the review in the context of what is already known, including mention of why a network meta-analysis has been conducted.	
Objectives	4	Provide an explicit statement of questions being addressed with reference to participants, interventions, comparisons, outcomes, and study design (PICOS).	
**METHODS**			
Protocol and registration	5	Indicate if a review protocol exists, if and where it can be accessed (e.g., Web address), and, if available, provide registration information including registration number.	
Eligibility criteria	6	Specify study characteristics (e.g., PICOS, length of follow-up) and report characteristics (e.g., years considered, language, publication status) used as criteria for eligibility, giving rationale. Clearly describe eligible treatments included in the treatment network, and note whether any have been clustered or merged into the same node (with justification).	
Information sources	7	Describe all information sources (e.g., databases with dates of coverage, contact with study authors to identify additional studies) in the search and date last searched.	
Search	8	Present full electronic search strategy for at least one database, including any limits used, such that it could be repeated.	
Study selection	9	State the process for selecting studies involved screening, eligibility, and determining which studies would be included in the meta-analysis).	
Data collection process	10	Describe method of data extraction from reports (e.g., piloted forms, independently, in duplicate) and any processes for obtaining and confirming data from investigators.	
Data items	11	List and define all variables for the research project or study. 3. Assumptions: Any underlying beliefs or suppositions that were made during the data were sought (e.g., PICOS, funding sources) and any assumptions and simplifications made.	
Geometry of the network	S1	Describe methods used to explore the geometry of the treatment network under study and potential biases related to it. This should include how the evidence base has been graphically summarized for presentation, and what characteristics were compiled and used to describe the evidence base to readers.	
Risk of bias in individual studies	12	Describe methods used for assessing risk of bias of individual studies (including specification of whether this was done at the study or outcome level), and how this information is to be used in any data synthesis.	
Summary measures	13	State the principal summary measures (e.g., risk ratio, difference in means). Also, describe the use of additional summary measures assessed, such as treatment rankings and surface under the cumulative	
		ranking curve (SUCRA) values, as well as modified approaches used to present summary findings from meta-analyses	
Synthesis of results	14	Describe the methods of handling data and combining results of studies for each network meta-analysis. This should include, but not be limited to: Handling of multi-arm trials; Selection of variance structure; Selection of prior distributions in Bayesian analyses; and Assessment of model fit.	
Assessment Of Inconsistency	S2	Describe the statistical methods used to evaluate the agreement of direct and indirect evidence in the treatment network(s) studied. Describe efforts taken to address its presence when found.	
Risk of bias across studies	15	Specify any assessment of risk of bias that may affect the cumulative evidence (e.g., publication bias, selective reporting within studies).	
Additional analyses	16	Describe methods of additional analyses, if done, indicating which were pre-specified. This may include, but not be limited to, the following: Sensitivity or subgroup analyses; Meta-regression analyses; Alternative formulations of the treatment network; and Use of alternative prior distributions for Bayesian analyses (if applicable).	
**RESULTS**			
Study selection	17	Give numbers of studies were screened, assessed for eligibility, and included in the review. The reasons for exclusions at each stage were as follows, ideally with a flow diagram.	
Presentation of network structure	S3	Provide a network graph of the included studies to enable visualization of the geometry of the treatment network	
Summary of network geometry	S4	Provide a brief overview of characteristics of the treatment network. This may include commentary on the abundance of trials and randomized patients for the different interventions and pairwise comparisons in the network, gaps of evidence in the treatment network, and potential biases reflected by the network structure	
Study characteristics	18	For each study, present characteristics for which data were extracted (e.g., study size, PICOS, follow-up period) and provide the citations.	
Risk of bias within studies	19	Present data on the risk of bias of each study and, if available, any outcome level assessment (see item 12).	
Results of individual studies	20	For all outcomes considered (benefits or harms), present, for each study: 1) simple summary data for each intervention group, and 2) effect estimates and confidence/credible intervals. Modified approaches may be needed to deal with information from larger networks.	
Synthesis of results	21	Present results of each meta-analysis done, including confidence/credible intervals. In larger networks, authors may focus on comparisons versus a particular comparator (e.g., placebo or standard care), with full findings presented in an appendix. League tables and forest plots may be considered to summarize pairwise comparisons. If additional summary measures were explored (such as treatment rankings), these should also be presented.	
Exploration for inconsistency	S5	Describe results from investigations of inconsistency. This may include such information as measures of model fit to compare consistent and inconsistency models, P values from statistical tests, or summary of inconsistency estimates from different parts of the treatment network.	
Risk of bias across studies	22	Present results of any assessment of risk of bias across studies (see Item 15)	
Additional analysis	23	Give results of additional analyses, if done (e.g., sensitivity or subgroup analyses, meta-regression, alternative network geometries studied, alternative choice of prior distributions for Bayesian analyses, and so forth [see Item 16]).	
**DISCUSSION**			
Summary of evidence	24	Summarize the main findings of this study can be summarized as follows: - There is strong evidence supporting the strength of the intervention in improving health outcomes. - The intervention was found to key groups (e.g., healthcare providers, users, and policy makers).	
Limitations	25	Discuss limitat theions at study and outcome level (e.g., risk of bias), and at the review level (e.g., incomplete retrieval of identified research, reporting bias). Comment on the validity of the assumptions, such as transitivity and consistency. Comment on any concerns regarding network geometry (e.g., avoidance of certain comparisons).	
Conclusions	26	Provide a general interpretation of the results in the context of other evidence and implications for future research.	
**FUNDING**			
Funding	27	Describe sources of funding for the systematic review and other support (e.g., supply of data); role of funders for the systematic review.	

PRISMA, Preferred Reporting Items for Systematic Reviews and Meta-Analysis; PICOS, population, intervention, comparators, outcomes, study design.

*Text in italics indicates wording specific to reporting of network meta-analyses that has been added to guidance from the PRISMA statement.

**Supplementary Table 2.** Literature Search Criteria

Search: (((Rectal Neoplasms[MeSH Terms]) OR (Neuroendocrine Tumors[MeSH Terms]) OR (Carcinoid Tumor[MeSH Terms]) OR (bleeding[Title/Abstract]) OR (perforations [Title/Abstract]) OR (stenosis[Title/Abstract]) OR (pneumonia[Title/Abstract]) OR (mucosal lacerations[Title/Abstract]) OR (Neoplasm, Rectal[Title/Abstract]) OR (Rectal Neoplasm[Title/Abstract]) OR (Rectum Neoplasms[Title/Abstract]) OR (Neoplasm, Rectum[Title/Abstract]) OR (Rectum Neoplasm[Title/Abstract]) OR (Rectal Tumors[Title/Abstract]) OR (Rectal Tumor[Title/Abstract]) OR (Tumor, Rectal[Title/Abstract]) OR (Neoplasms, Rectal[Title/Abstract]) OR (Cancer of Rectum[Title/Abstract]) OR (Rectum Cancers[Title/Abstract]) OR (Rectal Cancer[Title/Abstract]) OR (Cancer, Rectal[Title/Abstract]) OR (Rectal Cancers[Title/Abstract]) OR (Rectum Cancer[Title/Abstract]) OR (Cancer, Rectum[Title/Abstract]) OR (Cancer of the Rectum[Title/Abstract]) OR (Neuroendocrine Tumor[Title/Abstract]) OR (Tumor, Neuroendocrine[Title/Abstract]) OR (Tumors, Neuroendocrine[Title/Abstract]) OR (Carcinoid Tumors[Title/Abstract]) OR (Tumor, Carcinoid[Title/Abstract]) OR (Tumors, Carcinoid[Title/Abstract]) OR (Carcinoid[Title/Abstract]) OR (Carcinoids[Title/Abstract]) OR (Carcinoid, Goblet Cell[Title/Abstract]) OR (Carcinoids, Goblet Cell[Title/Abstract]) OR (Goblet Cell Carcinoid[Title/Abstract]) OR (Goblet Cell Carcinoids[Title/Abstract]) OR (Argentaffinoma[Title/Abstract]) OR (Argentaffinomas[Title/Abstract]) OR (rectal benign tumor[Title/Abstract]) OR (Benign tumor of the rectum[Title/Abstract]) OR (rectal benign neoplasm[Title/Abstract]) OR (Benign neoplasm of the rectum[Title/Abstract])) AND ((Endoscopic Mucosal Resection[MeSH Terms]) OR (Endoscopic Mucosal Resections[Title/Abstract]) OR (Mucosal Resection, Endoscopic[Title/Abstract]) OR (Resection, Endoscopic Mucosal[Title/Abstract]) OR (Strip Biopsy[Title/Abstract]) OR (Biopsy, Strip[Title/Abstract]) OR (Strip Biopsies[Title/Abstract]) OR (Endoscopic Mucous Membrane Resection[Title/Abstract]) OR (Endoscopic Submucosal Dissection[Title/Abstract]) OR (Dissection, Endoscopic Submucosal[Title/Abstract]) OR (Endoscopic Submucosal Dissections[Title/Abstract]) OR (Submucosal Dissection, Endoscopic[Title/Abstract]) OR (Endoscopic Full Thickness Resection[Title/Abstract]) OR (Submucosal Tunneling Endoscopic Resection[Title/Abstract]) OR (ESD[Title/Abstract]) OR (EMR[Title/Abstract]))) AND ((randomized controlled trial[Publication Type] OR randomized[Title/Abstract] OR placebo[Title/Abstract]) OR (incidence[MeSH:noexp] OR mortality[MeSH Terms] OR follow up studies[MeSH:noexp] OR prognos*[Text Word] OR predict*[Text Word] OR course*[Text Word])) Filters: from 2010 - 2023

**Supplementary Table 3.** Supplementary Quality Assessment of Individual Studies by the Newcastle-Ottawa Scale

Study	Selection				Comparability	Exposure			Scores
	Adequate definition of cases	Representativeness of the cases	Selection of controls	Definition of controls	Control for impotent factor	Ascertain-ment of exposure	Same method of ascertainment for cases and controls	Non-respons e rate	
Onozato et al^15^	★	★	★	★	★☆	★	★	★	8
Zhou et al^16^	★	★	☆	★	★☆	★	★	☆	6
Sung et al^17^	★	★	☆	★	☆☆	★	★	☆	5
Kim et al^18^	★	★	★	★	★★	★	★	☆	8
Niimi et al^19^	★	★	☆	★	★★	★	★	☆	7
Heo et al^20^	★	★	☆	★	★☆	★	★	★	7
Choi et al^21^	★	★	☆	★	★☆	★	★	★	7
Dou et al^22^	★	★	☆	★	☆☆	★	★	★	6
Lee et al^23^	★	★	☆	★	★☆	★	★	★	7
Jeon et al^24^	★	★	☆	★	★☆	★	★	★	7
Im et al^25^	☆	★	☆	★	☆☆	★	★	★	5
Huang et al^26^	★	★	★	★	★☆	★	★	★	8
Wang et al^27^	★	★	☆	★	★☆	★	★	★	7
Li ^28^	★	★	☆	★	★☆	★	★	☆	6
Cheung et al^29^	★	★	☆	★	★☆	★	★	★	7
Chen et al^30^	☆	★	☆	★	★★	★	★	★	7
Yang et al^31^	☆	★	☆	★	★★	★	★	☆	6
Bang et al^33^	☆	★	★	★	★☆	★	★	★	7
Choi et al^32^	☆	★	★	★	★☆	★	★	★	7
Zhang et al^34^	★	★	☆	★	★★	★	★	☆	7
Zhang et al^35^	☆	★	☆	★	★★	★	★	☆	6
Yang et al^36^	★	★	☆	★	★★	★	★	☆	7
Wang et al^37^	★	★	☆	★	★★	★	★	☆	7
Lim et al^38^	☆	★	★	★	★☆	★	★	☆	6
Shiet al^39^	☆	★	★	★	★☆	★	★	☆	6
Wu et al^40^	★	★	☆	★	★☆	★	★	★	7
Lee et al^41^	☆	★	★	★	★☆	★	★	☆	6
Wang et al^42^	★	★	★	★	★★	★	★	★	9
Park et al^43^	★	★	★	★	★★	★	★	☆	8
Wang et al^44^	★	★	☆	★	★☆	★	★	★	7
Liang ^45^	★	★	★	★	★☆	★	★	☆	7
Chen and Liang^46^	☆	★	★	★	★☆	★	★	☆	6
									
Wang et al^47^	☆	★	★	★	★☆	★	★	☆	6
Li et al^48^	★	★	☆	★	★☆	★	★	★	7
Hu^49^	★	★	★	★	★☆	★	★	☆	7
Jiang ^50^	☆	★	★	★	★☆	★	★	☆	6
Chen et al^51^	★	★	☆	★	★★	★	★	☆	7
Wu et al^52^	☆	★	★	★	★☆	★	★	☆	6

The Newcastle Ottawa Scale (NOS) was used to determine whether original studies were high quality (score 8 or 9), medium quality (score 6 or 7), or low quality (score 5).

**Supplementary Table 4.** Ranking Probabilities and SUCRA Values

Treatment	Rank of possibility									SUCRA	SUCRA (based on morphology)	SUCRA (based on histology)
	Best	2nd	3rd	4th	5th	6th	7th	8th	Worst			
**Histological Complete Resection (HCR)**												
EMR	0.00	0.00	0.00	0.00	0.00	0.00	0.04	0.19	**0.77**	**0.03**	**0.05**	**0.16**
EMRC	0.00	0.00	0.00	0.05	0.17	0.32	0.30	0.14	0.01	0.34	0.60	**0.91**
EMRL	0.00	0.04	0.60	0.29	0.05	0.01	0.00	0.00	0.00	0.70	**0.79**	0.82
EMRP	0.00	0.00	0.01	0.04	0.13	0.21	0.28	0.28	0.05	0.28	0.39	0.32
EMRD	0.00	0.01	0.03	0.09	0.11	0.16	0.23	0.28	0.09	0.29	0.62	0.17
EMRU	0.00	0.04	0.26	0.17	0.11	0.12	0.11	0.11	0.08	0.48	0.54	-
ESD	0.00	0.00	0.04	0.33	0.43	0.17	0.04	0.00	0.00	0.52	0.51	0.63
ESDM	0.40	0.54	0.04	0.01	0.01	0.01	0.01	0.00	0.00	0.91	-	-
TEM	**0.60**	0.37	0.02	0.00	0.00	0.00	0.00	0.00	0.00	**0.95**	-	-
**Surgery Time**												
EMR	0.15	0.36	0.26	0.16	0.05	0.02	0.00	0.00	0.00	0.79	0.74	**0.82**
EMRC	0.10	0.22	0.25	0.20	0.15	0.07	0.01	0.00	0.00	0.71	0.54	-
EMRL	0.01	0.06	0.18	0.30	0.31	0.13	0.01	0.00	0.00	0.63	0.60	0.61
EMRP	0.02	0.06	0.09	0.14	0.22	0.39	0.07	0.00	0.00	0.59	0.44	0.56
EMRD	0.13	0.15	0.13	0.13	0.17	0.22	0.07	0.00	0.00	0.51	0.41	-
EMRU	**0.58**	0.14	0.08	0.06	0.07	0.06	0.02	0.00	0.00	**0.86**	**0.77**	-
ESD	0.00	0.00	0.00	0.00	0.00	0.00	0.04	0.88	0.08	0.12	**0.00**	**0.01**
ESDM	0.00	0.00	0.01	0.01	0.04	0.11	0.76	0.05	0.00	0.28	-	-
TEM	0.00	0.00	0.00	0.00	0.00	0.00	0.01	0.07	**0.92**	**0.01**	-	-
**Complication**												
EMR	0.00	0.00	0.05	0.23	0.33	0.25	0.11	0.02	0.00	0.48	0.62	**0.62**
EMRC	0.00	0.01	0.09	0.30	0.27	0.18	0.10	0.03	0.01	0.50	0.59	0.56
EMRL	0.00	0.00	0.01	0.07	0.17	0.30	0.30	0.11	0.03	0.34	0.39	0.61
EMRP	0.00	0.00	0.01	0.04	0.06	0.10	0.17	0.26	0.34	0.18	0.32	0.36
EMRD	0.01	0.03	0.13	0.23	0.10	0.09	0.12	0.10	0.19	0.40	0.52	0.56
EMRU	0.28	0.26	0.26	0.06	0.03	0.03	0.03	0.02	0.04	0.77	**0.86**	-
ESD	0.00	0.00	0.00	0.00	0.00	0.03	0.16	0.44	**0.37**	**0.11**	**0.21**	**0.28**
ESDM	0.34	0.34	0.22	0.04	0.02	0.01	0.01	0.01	0.01	0.85	-	-
TEM	**0.37**	0.35	0.21	0.03	0.01	0.01	0.01	0.00	0.00	**0.87**	-	-

Measures with a higher probability of being ranked best and a higher SUCRA value had a higher HCR capacity, shorter surgery time, and fewer complications. Conversely, measures with a higher probability of being ranked worst and a lower SUCRA value had a weaker HCR capacity, took more surgery time, and had more complications. The first and last ranked results are in bold red.

**Supplementary Table 5.** Global Heterogeneity

Global heterogeneity			
Outcomes	Heterogeneity	Heterogeneity (based on morphology)	Heterogeneity (based on histology)
HCR	I2 = 2%	I2 = 5%	I2 = 9%
Surgery Time	I2 = 0%	I2 = 1%	I2 = 4%
Complication	I2 = 0%	I2 = 0%	I2 = 0%

Global heterogeneity was considered significant for I2 value ≥ 50%. There is no global heterogeneity in this network analysis and both two subgroup analyses as I2 is acceptable.

**Supplementary Table 6.** Local and Global Inconsistency

Inconsistency									
Comparisons	Main analysis			Subgroup (morphology)			Subgroup (histology)		
	Node-splitting	Consistency	Inconsistency	Node-splitting	Consistency	Inconsistency	Node-splitting	Consistency	Inconsistency
**Histological Complete Resection (HCR)**									
EMRC vs. EMR	*P* = .33	DIC = 145.50	DIC = 150.00	*P* = .91	DIC = 91.00	DIC = 91.40	*P* = .85	DIC = 67.20	DIC = 69.50
EMRD vs. EMR	*P* = .25			-			-		
EMRL vs. EMR	*P* = .69			*P* = 0.45			*P* = 0.46		
EMRP vs. EMR	*P* = .43			-			*P* = .14		
ESD vs. EMR	*P* = .66			*P* = .38			*P* = .25		
TEM vs. EMR	*P* = .71			-			-		
EMRL vs. EMRC	*P* = .59			*P* = .68			*P* = .79		
EMRP vs. EMRC	*P* = .71			-			-		
ESD vs. EMRC	*P* = .48			*P* = .61			-		
EMRP vs. EMRL	*P* = .68			-			-		
ESD vs. EMRL	*P* = .14			*P* = .09			*P* = .76		
ESD vs. EMRP	*P* = .79			-			*P* = .13		
**Surgery Time**									
EMRL vs. EMR	*P* = .051	DIC = 125.50	DIC = 126.40	*P* = .058	DIC = 75.50	DIC = 76.50	*P* = .15	DIC = 45.50	DIC = 45.70
ESD vs. EMR	***P* <.01**			***P* <.01**			*P* = .25		
EMRL vs. EMRC	*P* = .94			*P* = .88			-		
EMRP vs. EMRC	*P* = .94			-			-		
ESD vs. EMRC	*P* = .88			*P* = .99			-		
EMRP vs. EMRL	*P* = .94			-			-		
ESD vs. EMRL	*P* = .56			*P* = .98			*P* = .76		
**Complication**									
EMRC vs. EMR	*P* = .80	DIC = 98.70	DIC = 98.20	*P* = .91	DIC = 60.90	DIC = 59.50	*P* = .94	DIC = 38.60	DIC = 38.20
EMRD vs. EMR	*P* = .96			-			-		
EMRL vs. EMR	*P* = .41			*P* = .39			*P* = .65		
EMRP vs. EMR	***P* = .04**			-			*P* = .17		
ESD vs. EMR	*P* = .82			*P* = .33			*P* = .39		
EMRL vs. EMRC	*P* = .67			*P* = .58			*P* = .98		
ESD vs. EMRC	*P* = .52			*P* = .55			-		
ESD vs. EMRL	*P* = .60			*P* = .37			*P* = .64		
ESD vs. EMRP	*P* = .06			-			*P* = .24		

P-values of Node-splitting represent local inconsistency, and significant local inconsistency exists when the p-value <0.05. Deviance information criterion (DIC) represents global inconsistency, and significant global inconsistency exists when the difference between the DIC of the consistency model and the DIC of the inconsistency model > 5.00. Significant results are in bold red.

**Supplementary Table 7.** Results of network regression analyses for HCR, surgery time, and complication

Covariate	HCR		Surgery time		Complication	
	Articles (n)	β (95% CI)	Articles (n)	β (95% CI)	Articles (n)	β (95% CI)
En bloc resection rate	35	-1.50 (-3.90- 0.74)	27	2.24 (-15.46- 19.79)	30	-0.85 (-4.15- 2.25)
Clarify of surgery time	38	1.20 (-0.77- 3.27)	30	-0.15 (-13.82- 39.06)	33	1.21 (-0.74- 3.26)
Clarify of complication	38	-0.05 (-1.84- 1.88)	30	-3.01 (-43.95- 44.35)	33	-0.05 (-1.85- 1.89)
Publication year	38	0.67 (-1.08- 2.45)	30	38.80 (-16.60- 122.16)	33	0.66 (-1.08- 2.41)
Age	37	0.28 (-1.96- 2.74)	29	1.28 (-131.81- 57.46)	32	0.28 (-1.98- 2.76)
Sex	38	-0.94 (-3.32- 1.39)	30	-3.44 (-19.26- 17.47)	33	-0.94 (-3.35- 1.42)
Distance from anal verge	23	-1.77 (-5.44- 1.56)	18	5.74 (-19.10- 77.70)	18	-1.79 (-5.44- 1.53)

**Supplementary Table 8.** Summary of Confidence in Effect Estimates

Comparison	Nature of the evidence	Effect size	Within-study bias	Reporting bias	Indirectness	Heterogeneity	Incoherence	Confidence rating	Rating (based on morphology)	Rating (based on histology)
**Histological Complete Resection**										
EMRC:EMR	Mixed	1.30 (0.03, 2.49)	Some concerns	No concerns	No concerns	Major concerns	No concerns	Very low^1^	Low^12^	Low^12^
EMRL:EMR	Mixed	**2.62 (1.65, 3.66)**	Some concerns	No concerns	No concerns	No concerns	No concerns	Moderate^2^	Moderate^2^	Moderate^2^
EMRP:EMR	Mixed	1.10 (-0.45, 2.50)	Some concerns	No concerns	No concerns	No concerns	No concerns	Low	Low	Low
EMRD:EMR	Mixed	0.99 (-0.62, 2.83)	Some concerns	No concerns	No concerns	No concerns	No concerns	Low	Low	Very low^3^
EMRU:EMR	Indirect	1.91 (-1.00, 4.75)	Some concerns	No concerns	No concerns	No concerns	No concerns	Low	Low	-
ESD:EMR	Mixed	**1.87 (0.98, 2.71)**	Some concerns	No concerns	No concerns	Major concerns	No concerns	Low^12^	Low^1^^2^	Low^12^
ESDM:EMR	Indirect	**9.10 (1.93, 20.90)**	Some concerns	No concerns	No concerns	No concerns	No concerns	Moderate^2^	-	-
TEM:EMR	Mixed	**10.96 (3.06, 25.58)**	Some concerns	No concerns	No concerns	No concerns	No concerns	Moderate^2^	-	-
EMRL:EMRC	Mixed	1.32 (0.21, 2.50)	Some concerns	No concerns	No concerns	Major concerns	No concerns	Very low^1^	Low	Low
EMRP:EMRC	Mixed	-0.19 (-1.74, 1.27)	Some concerns	No concerns	No concerns	No concerns	No concerns	Low	Low	Low
EMRD:EMRC	Indirect	-0.31 (-2.17, 1.56)	Some concerns	No concerns	No concerns	No concerns	No concerns	Low	Low	Low
EMRU:EMRC	Indirect	0.61 (-2.27, 3.52)	No concerns	No concerns	No concerns	No concerns	No concerns	Low	Low	-
ESD:EMRC	Mixed	0.57 (-0.43, 1.63)	Some concerns	No concerns	No concerns	No concerns	No concerns	Low	Low	Low
ESDM:EMRC	Indirect	7.80 (0.64, 19.52)	Some concerns	No concerns	No concerns	No concerns	No concerns	Low	-	-
TEM:EMRC	Indirect	**9.66 (1.72, 24.15)**	Some concerns	No concerns	No concerns	No concerns	No concerns	Moderate^2^	-	-
EMRP:EMRL	Mixed	-1.52 (-3.05, -0.09)	Some concerns	No concerns	No concerns	Major concerns	No concerns	Very low^1^	Low	Low
EMRD:EMRL	Indirect	-1.63 (-3.37, 0.05)	Some concerns	No concerns	No concerns	No concerns	No concerns	Low	Low	Low^12^
EMRU:EMRL	Indirect	-0.71 (-3.74, 2.12)	Some concerns	No concerns	No concerns	No concerns	No concerns	Low	Low	-
ESD:EMRL	Mixed	-0.76 (-1.67, 0.09)	Some concerns	No concerns	No concerns	No concerns	No concerns	Low	Low	Low
ESDM:EMRL	Indirect	6.47 (-0.74, 18.30)	Some concerns	No concerns	No concerns	No concerns	No concerns	Low	-	-
TEM:EMRL	Indirect	8.33 (0.45, 22.99)	Some concerns	No concerns	No concerns	No concerns	No concerns	Low	-	-
EMRD:EMRP	Indirect	-0.12 (-2.01, 2.03)	Some concerns	No concerns	No concerns	No concerns	No concerns	Low	Low	Low
EMRU:EMRP	Indirect	0.81 (-2.30, 4.03)	No concerns	No concerns	No concerns	No concerns	No concerns	Low	Low	-
ESD:EMRP	Mixed	0.76 (-0.51, 2.07)	Some concerns	No concerns	No concerns	No concerns	No concerns	Low	Low	Low
ESDM:EMRP	Indirect	**7.99 (0.75, 19.91)**	Some concerns	No concerns	No concerns	Major concerns	No concerns	Low^12^	-	-
TEM:EMRP	Indirect	**9.85 (1.85, 24.48)**	Some concerns	No concerns	No concerns	No concerns	No concerns	Moderate^2^	-	-
EMRU:EMRD	Indirect	0.93 (-2.29, 4.21)	Some concerns	No concerns	No concerns	No concerns	No concerns	Low	Low	-
ESD:EMRD	Mixed	0.88 (-0.73, 2.44)	Some concerns	No concerns	No concerns	No concerns	No concerns	Low	Low	Very low^3^
ESDM:EMRD	Indirect	**8.11 (0.78, 19.70)**	Some concerns	No concerns	No concerns	Major concerns	No concerns	Low^12^	-	-
TEM:EMRD	Indirect	**9.97 (1.88, 24.81)**	Some concerns	No concerns	No concerns	No concerns	No concerns	Moderate^2^	-	-
ESD:EMRU	Direct	-0.05 (-2.81, 2.65)	No concerns	No concerns	No concerns	No concerns	No concerns	Low	Low	-
ESDM:EMRU	Indirect	7.18 (-0.70, 19.14)	Some concerns	No concerns	No concerns	No concerns	No concerns	Low	-	-
TEM:EMRU	Indirect	9.04 (0.51, 23.87)	Some concerns	No concerns	No concerns	No concerns	No concerns	Low	-	-
ESDM:ESD	Direct	7.23 (0.15, 18.97)	Some concerns	No concerns	No concerns	No concerns	No concerns	Low	-	-
TEM:ESD	Mixed	**9.09 (1.30, 23.78)**	Some concerns	No concerns	No concerns	No concerns	No concerns	Moderate^2^	-	-
TEM:ESDM	Indirect	1.86 (-12.96, 18.49)	Some concerns	No concerns	No concerns	No concerns	No concerns	Low	-	-
**Surgery Time**										
EMRC:EMR	Indirect	1.00 (-4.91, 7.02)	Some concerns	Major concerns	No concerns	No concerns	No concerns	Very low^4^	Very low^4^	-
EMRL:EMR	Mixed	2.37 (-2.10, 6.69)	Some concerns	Major concerns	No concerns	No concerns	No concerns	Very low^4^	Very low^4^	Very low^4^
EMRP:EMR	Indirect	3.90 (-3.05, 10.53)	Some concerns	Major concerns	No concerns	No concerns	No concerns	Very low^4^	Very low^4^	Very low^4^
EMRD:EMR	Indirect	2.18 (-7.24, 11.46)	Some concerns	Major concerns	No concerns	No concerns	No concerns	Very low^4^	Very low^4^	-
EMRU:EMR	Indirect	-2.71 (-13.76, 8.10)	Some concerns	Major concerns	No concerns	No concerns	No concerns	Very low^4^	Very low^4^	-
ESD:EMR	Mixed	**16.86 (12.62, 20.80)**	Some concerns	Major concerns	No concerns	No concerns	Major concerns	Very low ^245^	Very low^245^	Very low^124^
ESDM:EMR	Indirect	**10.12 (0.99, 18.38)**	Some concerns	Major concerns	No concerns	Major concerns	No concerns	Very low^124^	-	-
TEM:EMR	Mixed	**25.21 (13.57, 36.35)**	Some concerns	Major concerns	No concerns	No concerns	No concerns	Low^24^	-	-
EMRL:EMRC	Mixed	1.37 (-4.01, 6.63)	Some concerns	Major concerns	No concerns	No concerns	No concerns	Very low^4^	Very low^4^	-
EMRP:EMRC	Mixed	2.90 (-4.48, 10.15)	No concerns	Major concerns	No concerns	No concerns	No concerns	Very low^4^	Very low^4^	-
EMRD:EMRC	Indirect	1.18 (-8.56, 10.85)	Some concerns	Major concerns	No concerns	No concerns	No concerns	Very low^4^	Very low^4^	-
EMRU:EMRC	Indirect	-3.71 (-15.45, 7.61)	No concerns	Major concerns	No concerns	No concerns	No concerns	Very low^4^	Very low^4^	-
ESD:EMRC	Mixed	**15.86 (10.98, 20.49)**	Some concerns	Major concerns	No concerns	No concerns	No concerns	Low^24^	Low^24^	-
ESDM:EMRC	Indirect	9.12 (-0.01, 18.04)	Some concerns	Major concerns	No concerns	No concerns	No concerns	Very low^4^	-	-
TEM:EMRC	Indirect	**24.2 (10.93, 35.95)**	Some concerns	Major concerns	No concerns	No concerns	No concerns	Low^24^	-	-
EMRP:EMRL	Mixed	1.53 (-5.09, 7.94)	Some concerns	Major concerns	No concerns	No concerns	No concerns	Very low^4^	Very low^4^	Very low^4^
EMRD:EMRL	Indirect	-0.19 (-9.6, 9.17)	Some concerns	Major concerns	No concerns	No concerns	No concerns	Very low^4^	Very low^4^	-
EMRU:EMRL	Indirect	-5.08 (-16.06, 5.74)	No concerns	Major concerns	No concerns	No concerns	No concerns	Very low^4^	Very low^4^	-
ESD:EMRL	Mixed	**14.49 (10.95, 17.9)**	Some concerns	Major concerns	No concerns	No concerns	No concerns	Low^24^	Low^24^	Very low^124^
ESDM:EMRL	Indirect	7.75 (-1.28, 16.13)	Some concerns	Major concerns	No concerns	No concerns	No concerns	Very low^4^	-	-
TEM:EMRL	Indirect	**22.83 (10.71, 34.16)**	Some concerns	Major concerns	No concerns	No concerns	No concerns	Low^24^	-	-
EMRD:EMRP	Indirect	-1.72 (-12.00, 8.55)	Some concerns	Major concerns	No concerns	No concerns	No concerns	Very low^4^	Very low^4^	-
EMRU:EMRP	Indirect	-6.61 (-18.67, 5.22)	No concerns	Major concerns	No concerns	No concerns	No concerns	Very low^4^	Very low^4^	-
ESD:EMRP	Mixed	**12.96 (7.26, 19.05)**	Some concerns	Major concerns	No concerns	Major concerns	No concerns	Very low^124^	Very low^124^	Very low^124^
ESDM:EMRP	Indirect	6.22 (-3.84, 16.00)	Some concerns	Major concerns	No concerns	No concerns	No concerns	Very low^4^	-	-
TEM:EMRP	Indirect	**21.31 (7.74, 33.87)**	Some concerns	Major concerns	No concerns	No concerns	No concerns	Low^24^	-	-
EMRU:EMRD	Indirect	-4.89 (-17.98, 8.71)	No concerns	Major concerns	No concerns	No concerns	No concerns	Very low^4^	Very low^4^	-
ESD:EMRD	Direct	**14.68 (6.20, 23.31)**	Some concerns	Major concerns	No concerns	Major concerns	No concerns	Very low^124^	Very low^124^	-
ESDM:EMRD	Indirect	7.94 (-3.80, 19.35)	Some concerns	Major concerns	No concerns	No concerns	No concerns	Very low^4^	-	-
TEM:EMRD	Indirect	**23.03 (8.04, 36.62)**	Some concerns	Major concerns	No concerns	No concerns	No concerns	Low^24^	-	-
ESD:EMRU	Direct	**19.57 (8.80, 30.22)**	No concerns	Major concerns	No concerns	No concerns	No concerns	Low^24^	Low^24^	-
ESDM:EMRU	Indirect	12.83 (-0.46, 26.18)	Some concerns	Major concerns	No concerns	No concerns	No concerns	Very low^4^	-	-
TEM:EMRU	Indirect	**27.92 (11.68, 43.34)**	Some concerns	Major concerns	No concerns	No concerns	No concerns	Low^24^	-	-
ESDM:ESD	Direct	-6.75 (-14.83, 1.08)	Some concerns	Major concerns	No concerns	No concerns	No concerns	Very low^4^	-	-
TEM:ESD	Mixed	8.34 (-3.38, 19.33)	Some concerns	Major concerns	No concerns	No concerns	No concerns	Very low^4^	-	-
TEM:ESDM	Indirect	**15.09 (1.40, 28.50)**	Some concerns	Major concerns	No concerns	Major concerns	No concerns	Very low^124^	-	-
**Complication**										
EMRC:EMR	Mixed	-0.16 (-2.48, 2.14)	Some concerns	No concerns	No concerns	No concerns	No concerns	Low	Low	Low
EMRL:EMR	Mixed	0.65 (-1.01, 2.63)	Some concerns	No concerns	No concerns	No concerns	No concerns	Low	Low	Low
EMRP:EMR	Mixed	1.63 (-1.10, 4.86)	Some concerns	No concerns	No concerns	No concerns	Major concerns	Very low^5^	Low	Very low^3^
EMRD:EMR	Mixed	0.12 (-4.88, 5.36)	Some concerns	No concerns	No concerns	No concerns	No concerns	Low	Low	Low
EMRU:EMR	Indirect	-6.71 (-21.91, 3.31)	Some concerns	No concerns	No concerns	No concerns	No concerns	Low	Low	-
ESD:EMR	Mixed	1.79 (0.18, 3.79)	Some concerns	No concerns	No concerns	Major concerns	No concerns	Very low^1^	Low	Low
ESDM:EMR	Indirect	-8.26 (-22.4, 1.15)	Some concerns	No concerns	No concerns	No concerns	No concerns	Low	-	-
TEM:EMR	Mixed	-8.82 (-23.13, 0.25)	Some concerns	No concerns	No concerns	No concerns	No concerns	Low	-	-
EMRL:EMRC	Mixed	0.81 (-1.33, 3.42)	Some concerns	No concerns	No concerns	No concerns	No concerns	Low	Low	Low
EMRP:EMRC	Indirect	1.79 (-1.46, 5.36)	Some concerns	No concerns	No concerns	No concerns	No concerns	Low	Low	Low
EMRD:EMRC	Indirect	0.28 (-4.77, 5.81)	Some concerns	No concerns	No concerns	No concerns	No concerns	Low	Low	Low
EMRU:EMRC	Indirect	-6.55 (-21.75, 3.54)	Some concerns	No concerns	No concerns	No concerns	No concerns	Low	Low	-
ESD:EMRC	Mixed	1.95 (0.01, 4.51)	Some concerns	No concerns	No concerns	Major concerns	No concerns	Very low^1^	Low	Low
ESDM:EMRC	Indirect	-8.10 (-22.28, 1.68)	Some concerns	No concerns	No concerns	No concerns	No concerns	Low	-	-
TEM:EMRC	Indirect	-8.66 (-23.16, 0.61)	Some concerns	No concerns	No concerns	No concerns	No concerns	Low	-	-
EMRP:EMRL	Indirect	0.97 (-2.05, 4.01)	Some concerns	No concerns	No concerns	No concerns	No concerns	Low	Low	Low
EMRD:EMRL	Indirect	-0.53 (-5.63, 4.58)	Some concerns	No concerns	No concerns	No concerns	No concerns	Low	Low	Low
EMRU:EMRL	Indirect	-7.36 (-22.8, 2.63)	Some concerns	No concerns	No concerns	No concerns	No concerns	Low	Low	-
ESD:EMRL	Mixed	1.14 (-0.49, 2.88)	Some concerns	No concerns	No concerns	No concerns	No concerns	Low	Low	Low
ESDM:EMRL	Indirect	-8.92 (-23.13, 0.44)	Some concerns	No concerns	No concerns	No concerns	No concerns	Low	-	-
TEM:EMRL	Indirect	-9.48 (-23.82, -0.32)	Some concerns	No concerns	No concerns	Major concerns	No concerns	Very low^1^	-	-
EMRD:EMRP	Indirect	-1.50 (-6.91, 3.85)	Some concerns	No concerns	No concerns	No concerns	No concerns	Low	Low	Low
EMRU:EMRP	Indirect	-8.33 (-23.74, 2.05)	No concerns	No concerns	No concerns	No concerns	No concerns	Low	Low	-
ESD:EMRP	Mixed	0.17 (-2.54, 2.92)	Some concerns	No concerns	No concerns	No concerns	No concerns	Low	Low	Low
ESDM:EMRP	Indirect	-9.89 (-24.23, -0.34)	Some concerns	No concerns	No concerns	Major concerns	No concerns	Very low^1^	-	-
TEM:EMRP	Indirect	**-10.45 (-25.26, -1.02)**	Some concerns	No concerns	No concerns	No concerns	No concerns	Moderate^2^	-	-
EMRU:EMRD	Indirect	-6.83 (-22.27, 4.28)	Some concerns	No concerns	No concerns	No concerns	No concerns	Low	Low	-
ESD:EMRD	Mixed	1.67 (-3.21, 6.64)	Some concerns	No concerns	No concerns	No concerns	No concerns	Low	Low	Very low^3^
ESDM:EMRD	Indirect	-8.39 (-23.1, 2.15)	Some concerns	No concerns	No concerns	No concerns	No concerns	Low	-	-
TEM:EMRD	Indirect	-8.95 (-23.68, 1.85)	Some concerns	No concerns	No concerns	No concerns	No concerns	Low	-	-
ESD:EMRU	Direct	8.50 (-1.31, 23.58)	No concerns	No concerns	No concerns	No concerns	No concerns	Low	Low	-
ESDM:EMRU	Indirect	-1.56 (-19.45, 17.35)	Some concerns	No concerns	No concerns	No concerns	No concerns	Low		
TEM:EMRU	Indirect	-2.12 (-20.09, 15.95)	Some concerns	No concerns	No concerns	No concerns	No concerns	Low	-	-
ESDM:ESD	Direct	**-10.06 (-24.94, -1.66)**	Some concerns	No concerns	No concerns	No concerns	No concerns	Moderate^2^	-	-
TEM:ESD	Mixed	**-10.62 (-24.94, -1.66)**	Some concerns	No concerns	No concerns	No concerns	No concerns	Moderate^2^	-	-
TEM:ESDM	Indirect	-0.56 (-18.06, 16.28)	Some concerns	No concerns	No concerns	No concerns	No concerns	Low	-	-

The colors represent the risk of bias (green: No concerns or Low risk or High confidence, blue: Moderate confidence, yellow: Some concerns or Low confidence, red: Major concerns or Very low confidence).

^1^Confidence in evidence was downgraded for significant heterogeneity.

^2^Confidence in evidence was upgraded for large magnitude effect (OR >2 or MD >0).

^3^Confidence in evidence was downgraded for high risk study bias.

^4^Confidence in evidence was downgraded for reporting bias.

^5^Confidence in evidence was downgraded for inconsistency.

**Supplementary Table 9.** Glossary of Abbreviations

R-NENs: rectal neuroendocrine neoplasms EMR: endoscopic mucosal resection EMRM: modified endoscopic mucosal resection EMRC: endoscopic mucosal resection with cap EMRL: endoscopic mucosal resection with ligation EMRP: endoscopic mucosal resection with pre-cutting EMRD: endoscopic mucosal resection with dual-channel endoscope EMRU: endoscopic mucosal resection under water ESD: endoscopic submucosal dissection ESDM: modified endoscopic submucosal dissection TEM: transanal endoscopic microsurgery NMA: network meta-analysis PRISMA-NMA: preferred reporting items for systematic reviews and meta-analyses extension statement for network meta-analysis PROSPERO: prospective register of systematic reviews HCR: histological complete resection OR: odds ratio MD: mean difference SUCRA: the surface under the cumulative ranking curve 95% CI: 95% confidence interval 95% PrI: 95% confidence interval DIC: deviance information criterion

**Supplementary Table 10.** Information About the Outcome Events in Studies

Author, Year	Sex (Male/Female)	Age, year (Mean)	Group Comparisons	No. of Lesions	Distance from anal verge, cm (Mean)	No. of Histological Complete Resection	No. of Endoscopic Complete Resection	Surgery Time, min (Mean ± SD)	No. of Complication	En-bloc resection rate (%)	Clarify of surgery time (%)	Clarify of complication (%)
**Onozatoet al^15^**	3.13	N/A	A: EMRD	26	N/A	22	26	9.3 ± 2.2	0	N/A	0	0
	3.13	N/A	B: ESD	9	N/A	7	9	25.6 ± 8.8	0	N/A	0	0
**Zhou et al^16^**	1.53	48.95	A: EMR	23	N/A	12	20	12.3 ± 15.4	0	93	1	1
	1.53	48.95	B: ESD	20	N/A	20	20	28.4 ± 17.2	1	93	1	1
**Sung et al^17^**	1.66	52.3	A: EMR	14	N/A	10	14	N/A	0	100	0	0
	1.66	52.3	B: EMRD	58	N/A	43	58	N/A	0	100	0	0
	1.66	52.3	C: ESD	5	N/A	5	5	N/A	0	100	0	0
**Kimet al^18^**	1.94	51.15	A: EMRL	45	7.7	42	45	4.8 ± 0.9	2	95	0	1
	1.94	51.15	B: EMR	55	7.7	36	50	5.0 ± 0.8	0	95	0	1
**Niimi et al^19^**	2.43	50.4	A: ESD	13	N/A	12	13	28.6 ± 16.2	0	100	1	1
	2.43	50.4	B: EMRL	11	N/A	11	11	17.4 ± 4.4	1	100	1	1
**Heoet al^20^**	1.61	49.8	A: EMRL	48	N/A	46	48	7.6 ± 3.2	2	97.2	0	1
	1.61	49.8	B: EMR	34	N/A	30	32	3.9 ± 1.6	1	97.2	0	1
**Choi et al^21^**	1.40	48.03	A: EMRL	29	8.13	24	29	6.4 ± 3.5	N/A	100	0	1
	1.40	48.03	B: ESD	31	8.13	25	31	15.1 ± 5.7	N/A	100	0	1
**Douet al^22^**	1.39	48.8	A: EMR	26	N/A	26	26	8.9 ± 6.3	5	100	0	0
	1.39	48.8	B: ESD	20	N/A	19	20	32.6 ± 10.5	1	100	0	0
**Lee et al^23^**	2.04	49.4	A: EMRD	44	6.8	38	44	9.8 ± 7.1	1	100	1	1
	2.04	49.4	B: ESD	26	6.8	23	26	22.4 ± 7.6	2	100	1	1
**Jeon et al^24^**	2.47	49	A: EMR	29	6.5	19	29	6.5 ± 3.6	5	100	0	0
	2.47	49	B: ESD	23	6.5	19	23	18.0 ± 13.2	11	100	0	0
	2.47	49	C: TEM	14	6.5	14	14	40.7 ± 14.2	0	100	0	0
**Im et al^25^**	1.66	50.65	A: EMRL	35	7.4	33	35	4.2 ± 1.5	1	95.4	1	1
	1.66	50.65	B: ESD	74	7.4	56	69	5.1 ± 2.5	3	95.4	1	1
**Huang et al^26^**	1.19	49.5	A: EMRP	31	6.15	30	31	N/A	31	98.4	1	0
	1.19	49.5	B: EMR	28	6.15	23	27	N/A	21	98.4	1	0
**Wang et al^27^**	1.20	44.86	A: EMRC	30	7.39	21	25	9.5 ± 2.1	0	90.9	1	1
	1.20	44.86	B: ESD	25	7.39	25	25	24.8 ± 4.9	3	90.9	1	1
**Li^28^**	1.27	52	A: EMR	35	6.2	27	N/A	N/A	2	88.3	0	0
	1.27	52	B: EMRC	42	6.2	41	N/A	N/A	3	88.3	0	0
**Cheunget al^29^**	3.71	48.8	A: ESD	17	6.76	10	17	20.2 ± 12.6	1	93.9	1	1
	3.71	48.8	B: EMRP	16	6.76	7	14	9.7 ± 3.6	1	93.9	1	1
**Chen et al^30^**	1.26	51.25	A: EMRP	33	8.17	31	32	25.7 ± 11.7	2	98.4	1	0
	1.26	51.25	B: ESD	28	8.17	27	28	41.7 ± 21.2	5	98.4	1	0
**Yang et al^31^**	1.30	50	A: EMRL	27	N/A	25	27	15.6 ± 6.3	3	100	0	0
	1.30	50	B: ESD	19	N/A	18	19	28.7 ± 10.6	4	100	0	0
**Bang et al^33^**	1.85	52.7	A: EMRL	53	5.5	53	53	5.3 ± 2.8	2	100	0	1
	1.85	52.7	B: ESD	24	5.5	13	24	17.9 ± 9.1	0	100	0	1
**Choi et al^32^**	1.85	50.8	A: EMRC	65	N/A	59	65	N/A	0	100	0	1
	1.85	50.8	B: EMRL	16	N/A	13	16	N/A	0	100	0	1
**Zhang et al^34^**	0.94	47.7	A: EMRP	30	6.32	23	29	8.5 ± 3.4	0	100	1	1
	0.94	47.7	B: ESD	36	6.32	35	36	20.4 ± 6.6	1	100	1	1
**Zhang et al^35^**	1.26	46.11	A: EMRC	29	6.04	25	N/A	15.4 ± 2.8	0	88.5	0	0
	1.26	46.11	B: ESD	23	6.04	21	N/A	29.0 ± 3.6	2	88.5	0	0
**Yang et al^36^**	1.33	49	A: EMRC	27	N/A	24	25	5.8 ± 1.3	2	95.2	1	0
	1.33	49	B: ESD	15	N/A	13	15	31.4 ± 8.1	2	95.2	1	0
**Wang et al^37^**	1.88	56.48	A: EMR	22	N/A	16	N/A	1.6 ± 0.1	2	N/A	0	0
	1.88	56.48	B: EMRL	20	N/A	19	N/A	2.0 ± 0.1	1	N/A	0	0
	1.88	56.48	C: ESD	7	N/A	7	NA	31.9 ± 0.4	0	N/A	0	0
**Limet al^38^**	1.22	51.8	A: EMRL	66	6.25	63	66	7.1 ± 4.5	0	100	0	1
	1.22	51.8	B: ESD	16	6.25	12	16	24.2 ± 12.2	0	100	0	1
**Shi et al^39^**	1.64	56	A: ESDM	17	5.35	17	17	14.7 ± 3.3	0	100	1	0
	1.64	56	B: ESD	20	5.35	20	20	17.9 ± 6.6	0	100	1	0
**Wu et al^40^**	1.26	51	A: EMRL	23	N/A	22	23	15.4 ± 4.7	1	100	1	0
	1.26	51	B: ESD	20	N/A	20	20	20.7 ± 6.4	1	100	1	0
**Lee et al^41^**	1.36	51	A: EMRC	42	7.8	35	39	5.5 ± 2.5	2	98.1	1	0
	1.36	51	B: EMRL	120	7.8	111	120	5.5 ± 5.9	5	98.1	1	0
**Wang et al^42^**	1.56	49.49	A: EMRC	23	N/A	19	23	9.6 ± 4.8	N/A	98.8	1	1
	1.56	49.49	B: EMRL	26	N/A	26	26	8.5 ± 5.1	0	98.8	1	1
	1.56	49.49	C: EMRP	30	N/A	27	29	9.2 ± 3.5	N/A	98.8	1	1
	1.56	49.49	D: ESD	259	N/A	238	256	17.2 ± 9.7	7	98.8	1	1
**Park et al^43^**	1.67	46.35	A: EMRU	36	6.25	31	N/A	5.8 ± 2.9	0	N/A	0	0
	1.67	46.35	B: ESD	79	6.25	68	N/A	26.6 ± 13.4	2	N/A	0	0
**Wang et al^44^**	2.00	50.3	A: ESD	28	7.08	27	26	35.1 ± 7.2	N/A	95.7	1	0
	2.00	50.3	B: EMR	41	7.08	25	40	5.7 ± 1.1	N/A	95.7	1	0
**Liang ^45^**	1.98	44.2	A: EMRC	94	7.11	77	94	10.5 ± 9.0	2	100	0	1
	1.98	44.2	B: ESD	67	7.11	55	67	28.4 ± 9.8	7	100	0	1
**Chen and Liang^46^**	1.41	50.4	A: EMRC	31	6.82	29	29	N/A	N/A	92.3	0	0
	1.41	50.4	B: ESD	34	6.82	31	31	N/A	N/A	92.3	0	0
**Chen and Liang 46**	1.25	48.55	A: EMRC	20	6.4	13	11	N/A	N/A	77.8	0	0
	1.25	48.55	B: ESD	25	6.4	24	24	N/A	N/A	77.8	0	0
**Wang et al^47^**	0.91	51.4	A: ESD	76	6.6	69	76	N/A	N/A	100	0	0
	0.91	51.4	B: TEM	35	6.6	35	35	N/A	N/A	100	0	0
**Li et al^48^**	0.83	54.85	A: ESD	21	N/A	21	21	29.9 ± 3.81	0	100	0	0
	0.83	54.85	B: EMRL	21	N/A	21	21	10.1 ± 1.37	0	100	0	0
**Hu^49^**	1.25	48.2	A: EMR	50	N/A	39	50	N/A	N/A	86.7	0	0
	1.25	48.2	B: ESD	40	N/A	39	40	N/A	N/A	86.7	0	0
**Jiang^50^**	0.89	50.4	A: EMRL	28	6.24	28	28	N/A	0	100	0	1
	0.89	50.4	B: ESD	31	6.24	24	31	N/A	13	100	0	1
	0.89	50.4	C: EMR	9	6.24	4	9	N/A	0	100	0	1
**Chen et al^51^**	1.15	51.05	A: EMRL	26	6.3	24	26	9.08 ± 3.45	0	92.9	0	0
	1.15	51.05	B: ESD	30	6.3	28	30	18.5 ± 3.25	1	92.9	0	0
** Wu et al^52^**	1.29	47.1	A: ESDC	28	N/A	28	28	13.8 ± 4.2	0	100	0	0
	1.29	47.1	B: ESD	27	N/A	24	27	19.9 ± 3.9	6	100	0	0

EMR, endoscopic mucosal resection; EMRC, endoscopic mucosal resection with cap; EMRL, endoscopic mucosal resection with ligation; EMRP, endoscopic mucosal resection with pre-cutting; EMRD, endoscopic mucosal resection with dual-channel endoscope; EMRU, endoscopic mucosal resection under water; ESD, endoscopic submucosal dissection; ESDM, modified endoscopic submucosal dissection; TEM, transanal endoscopic microsurgery; NA, not available.

^1^Two subgroups in the same study
